# Electronic Structure Engineering of Single‐Atom Tungsten on Vacancy‐enriched V_3_S_4_ Nanosheets for Efficient Hydrogen Evolution

**DOI:** 10.1002/advs.202409855

**Published:** 2024-10-28

**Authors:** Min Xi, Hua Zhang, Lingfeng Yang, Youyu Long, Yifan Zhao, Anran Chen, Qiaozhi Xiao, Tingting Liu, Xuechun Xiao, Guangzhi Hu

**Affiliations:** ^1^ Yunnan Key Laboratory of Electromagnetic Materials and Devices School of Materials and Energy Yunnan University Kunming 650091 China; ^2^ Electron Microscopy Center Yunnan University Kunming 650091 China; ^3^ Institute for Ecological Research and Pollution Control of Plateau Lakes School of Ecology and Environmental Science Yunnan University Kunming 650091 China

**Keywords:** electronic structure, hydrogen evolution reaction, single‐atom catalyst, sulfur vacancy, transition metal sulfide

## Abstract

Constructing single‐atom catalysts (SACs) and optimizing the electronic structure between metal atoms and support interactions is deemed one of the most effective strategies for boosting the catalytic kinetics of the hydrogen evolution reaction (HER). Herein, a sulfur vacancy defect trapping strategy is developed to anchor tungsten single atoms onto ultrathin V_3_S_4_ nanosheets with a high loading of 25.1 wt.%. The obtained W‐V_3_S_4_ catalyst exhibits a low overpotential of 54 mV at 10 mA cm^−2^ and excellent long‐term stability in alkaline electrolytes. Density functional theory calculations reveal that the in situ anchoring of W single atoms triggers the delocalization and redistribution of electron density, which effectively accelerates water dissociation and facilitates hydrogen adsorption/desorption, thus enhancing HER activity. This work provides valuable insights into understanding highly active single‐atom catalysts for large‐scale hydrogen production.

## Graphical abstract

The sulfur vacancy defect trapping strategy to anchor tungsten single atoms onto ultrathin V _3_ S_4_ nanosheets with a high loading of 25.1 wt.%. The in situ anchoring of W single atoms successfully triggers the delocalization and redistribution of electron density of V_3_S_4_, which effectively accelerates water dissociation and facilitates hydrogen adsorption/desorption, resulting in excellent catalytic performance and long‐term stability.

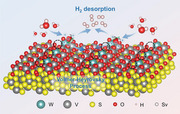

## 1 Introduction

The proliferation of fossil fuel consumption leads to irreversible environmental consequences globally, highlighting the significance of efficient and sustainable energy‐support technologies in the development of renewable energy sources. Hydrogen (H_2_) is considered a promising alternative energy source to address the current energy crisis and mitigate severe environmental pollution due to its high energy density and eco‐friendliness (generating only water upon combustion).^[^ ^1^ ^]^ Electrochemical water splitting presents unique advantages for producing large‐scale, high‐purity hydrogen compared to other feasible strategies.^[^ ^2^ ^]^ However, the water dissociation process of the hydrogen evolution reaction (HER) in alkaline environments is enslaved by the thermodynamic energy barrier inherent in the Volmer step (H_2_O + e^−^ → H_ad_ + OH^−^).^[^ ^3^ ^]^ Thus, a critical evaluation of the balance between HO─H bond cleavage, H adsorption, and OH desorption is imperative for a wide range of alkaline HER electrocatalysts.^[^ ^4^ ^]^ Meanwhile, despite their exceptional catalytic activity, the scarcity of reserves and the expensive overall cost of producing high‐purity hydrogen render traditional Pt‐based catalysts impractical for commercial applications.^[^ ^5^ ^]^ Consequently, the quest for efficient and robust non‐noble metal electrocatalysts capable of accelerating water dissociation with outstanding activity remains a formidable challenge.^[^ ^6^ ^]^

Single‐atom catalysts (SACs) offer potential advantages such as an unsaturated coordination environment, high catalytic selectivity, and maximum atomic utilization efficiency. Tungsten (W) is a promising candidate for SACs due to its unique properties, including a positive charge, large spin magnetic moment, and excellent electrical conductivity.^[^ ^7^ ^]^ Strong metal‐support interactions (MSIs) facilitate the formation of thermodynamically favorable metal‐support bonds, effectively modifying the electronic structure of the catalyst while stabilizing single metal atoms by anchoring structural defects associated with the unsaturated sites.^[^ ^8^ ^]^ Furthermore, electron transfer induces charge redistribution, which not only adjusts the d‐orbital electrons of the catalyst but also facilitates intermediate adsorption and accelerates the rate‐limiting step.^[^ ^9^ ^]^ However, the loading capacity of metal atoms is typically kept low (less than 1.5 wt.%) to prevent agglomeration resulting from their high surface energy. Additionally, inevitable active center poisoning or deactivating occurs, especially in harsh alkaline environments, caused by the relatively weak interaction between SAs and catalyst substrate.^[^ ^10^ ^]^ Li et al. prepare well‐defined isolated Pt single atoms on nitrogen‐doped porous carbon (Pt_1_/NPC) with a loading of 3.8 wt.%, the excellent HER activity from the unique electronic structure of the Pt‐N_4_ ligand site in Pt_1_/NPC.^[^ ^11^ ^]^ Fu et al. synthesize Cu single‐atom catalysts (n‐Cu/BP) on BP nanosheets (NSs) with a loading of up to 11.3%. This catalyst, with its unique electron‐rich characteristics, significantly enhances hydrogen production efficiency in alkaline conditions.^[^ ^12^ ^]^ Therefore, constructing suitable matrices with high‐density thermal stability by exploiting defects to immobilize single‐atom catalysts plays an essential role in improving HER activities.

Recent studies demonstrate that transition metal dichalcogenides (TMDs) such as MoS_2_, NiS_2_, Co_9_S_8_, VS_2_, VS_4_, and V_3_S_4_ effectively enhance their adsorption capacity for hydrogen atoms and the structural stability of the central metal. This enhancement favors the electrocatalytic activity of the HER during water electrolysis due to their unique structural configurations.^[^ ^13^ ^]^ Vanadium sulfides, particularly V_3_S_4_ in the form of V_0.5_VS_2_, possess a unique aberrated 3D monoclinic NiAs‐type crystal structure with large interlayer spacing (0.569 nm) and specific conductivity. This structure consists of a VS_2_ monolayer with additional V atoms connecting adjacent layers, creating a flexible pillar‐layered arrangement. This configuration effectively provides multiple active sites for modulating catalytic activity.^[^ ^14^ ^]^ Previous literature investigates the combination of favorable characteristics of vanadium sulfides with single atoms to achieve a more tailored surface electronic structure through defect engineering. These defects act as metallic nodes in the flexible substances composed of controllably loaded masses of heterogeneous atoms and abundant vacancy defects, promoting localized electron redistribution and expediting the charge transfer rate.^[^ ^15^ ^]^ Li et al. developed nickel‐cobalt sulfide heterostructures (CN@NiCoS) encapsulated in nitrogen‐doped carbon shells, introducing sulfur vacancies at the heterointerfaces to enhance sulfur migration activity, resulting in long‐term stability of up to 1000 h.^[^ ^16^ ^]^ Introducing heteroatoms at the atomic level allows precise control over their location, inducing crystal distortion that modulates the band structure and valence state, thus optimizing intermediates' adsorption/desorption kinetics.^[^ ^17^ ^]^ Therefore, combining defect‐rich V_3_S_4_‐based catalysts with highly loaded metal atoms is expected to enhance alkaline HER performance through MISs and the resulting synergistic effects.^[^ ^13^, ^18^ ^]^

In this work, we successfully immobilized high‐loading W single atoms (W SAs) on the sulfur vacancies (S_V_) of the ultrathin V_3_S_4_ nanosheets via a hydrothermal and in situ anchoring synthesis method (W‐V_3_S_4_). The W‐V_3_S_4_ catalyst exhibits favorable HER catalytic activity (54 mV at 10 mA cm^−2^) and long‐term stability in alkaline electrolytes. Density functional theory calculations confirm that anchoring W SAs effectively induces a redistribution of charge density and optimizes the adsorption/desorption of intermediates, ultimately boosting the overall HER performance. This study presents an effective strategy for theoretical guidance to design efficient HER catalysts with high‐loading single‐atom catalysts.

## 2 Results and Discussion

### 2.1 Materials Characterization of Catalyst

**Figure 1:**
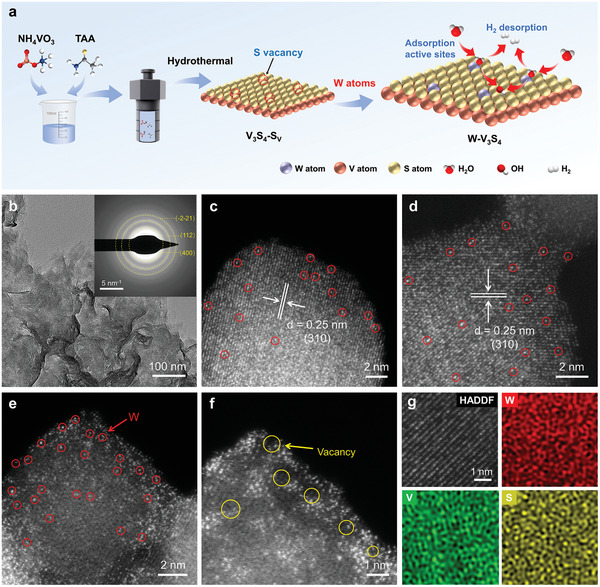
Structure analysis. a) Synthetic process schematic for W‐V_3_S_4_ catalysts. b) TEM images of W‐V_3_S_4_. c–f) HRTEM images of W‐V_3_S_4_. g) HAADF‐STEM image and EDS elemental mapping of W, V, and S elements for W‐V_3_S_4_.

**Figure** **1**a depicts the elaborate synthesis procedure of the W‐decorated V_3_S_4_ electrocatalyst. Briefly, the S‐vacancy‐rich V_3_S_4_ NSs were prepared via a simple and facile hydrothermal treatment. Then, the highly loaded W single atoms (SAs) were anchored onto the V_3_S_4_ substrate via in situ anchoring (refer to Supporting Information for experimental details). Transmission electron microscopy (TEM) analysis revealed the preliminary morphology of as‐prepared W‐V_3_S_4_ catalysts as uniform and ultrathin nanosheet structures without visible metal particles (Figure 1b). The inset in Figure 1b illustrates a polycrystalline structure in the corresponding selected area electron diffraction (SAED) pattern. This structure closely resembled that of pure V_3_S_4_ NSs (Figure S1, Supporting Information), characterized by the absence of interlayer stacking and uniform dispersion of V and S atoms, as confirmed by the corresponding energy spectroscopy (EDS) mapping analyses (Figure S2, Supporting Information). Notably, as shown in Figure 1c,d, the majority of the W SAs, identified by isolated bright sites marked by red circles, were uniformly dispersed on the V_3_S_4_ NSs, as observed by high‐angle annular dark‐field scanning transmission electron microscopy (HAADF‐STEM) imaging. The spacing of the lattice at 0.25 nm correlates with the (310) crystal plane of V_3_S_4_, with no discernible W nanoparticles or clusters detected at different magnifications, demonstrating the presence of isolated W SAs and S_V_ dispersed in the V_3_S_4_ substrate (Figure 1e,f; Figure S3, Supporting Information). In addition, the corresponding EDS maps, in conjunction with the HAADF‐STEM image, verified the uniform distribution of W, V, and S elements throughout the V_3_S_4_ NSs (Figure 1g).

The crystal structure of the W‐V_3_S_4_ electrocatalyst was unveiled through X‐ray diffraction (XRD) patterns (Figure S4, Supporting Information). The diffraction peaks matched well with the V_3_S_4_ standard data (JCPDS No. 18–1456), indicating successful doping of W into the V_3_S_4_ lattice without additional diffraction peaks corresponding to the W crystal phase. The W atomic loading was quantified as 25.1 wt.% via inductively coupled plasma optical emission spectroscopy (ICP‐OES) (Table S1, Supporting Information), consistent with results obtained from the EDS mapping. Raman spectroscopy measurements revealed nearly identical characteristic peaks before and after W anchoring (Figure S5, Supporting Information). Electron paramagnetic resonance (EPR) analysis, as shown in Figure S6 (Supporting Information), confirmed the presence of the S_V_ in the sample. The EPR spectrum of V_3_S_4_ exhibited a strong signal at g = 2.003, indicating an abundance of S_V_ and defects on the surface, which facilitated the attachment sites of single atoms. However, the slightly enhanced and broadened EPR spectrum of W‐V_3_S_4_ suggested a reduced distribution of S_V_, likely attributed to electrochemical etching during the annealing process, supporting the single‐atom state of W atoms. This observation was in agreement with HAADF‐STEM results.^[^ ^19^ ^]^ The N_2_ adsorption‐desorption isotherms and curves describing pore size distribution, analyzed via the Brunauer‐Emmentt‐Teller (BET) method (Figure S7, Supporting Information), revealed key characteristics of the W‐V_3_S_4_ electrocatalyst. Compared to V_3_S_4_, W‐V_3_S_4_ exhibited a greater specific surface area of 37.5 m^2^ g^−1^ and a wider pore diameter of 5.5 nm, surpassing V_3_S_4_ (37 m^2^ g^−1^ and 1.4 nm) and indicating the intrinsic open and porous structure of W‐V_3_S_4_, which facilitated better anchoring of W SAs.^[^ ^20^ ^]^ It also enhanced the contact area between the electrolyte and the catalyst, facilitated more effective electrolyte penetration, and accelerated the reaction kinetics via the abundant active sites.

**Figure 2:**
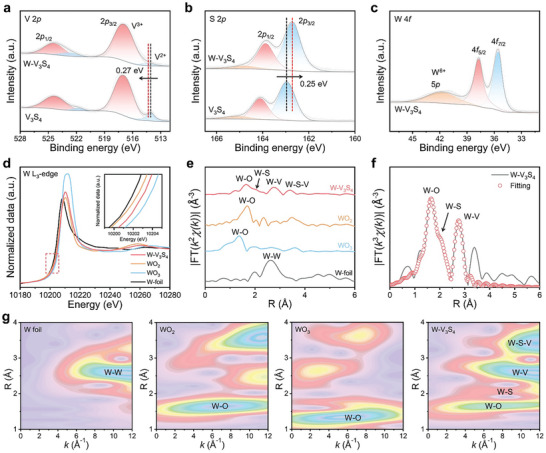
Analysis of the W‐V_3_S_4_ structure. a–c) High‐resolution XPS spectra of (a) V 2*p*, (b) S 2*p*, and (c)W 4*f* for W‐V_3_S_4_ and V_3_S_4_. d) W L_3_‐edge XANES spectra of W‐V_3_S_4_, WO_2_, WO_3_, W‐foil. e) R‐space W L_3_‐edge FT‐EXAFS spectra of W‐V_3_S_4_, WO_2_, WO_3_, W‐foil. f) The fitting curve of EXAFS R‐space of W‐V_3_S_4_. g) WT‐EXAFS signals of the W‐V_3_S_4_ and the references.

The surface chemical composition and valence states of both the W‐V_3_S_4_ and V_3_S_4_ catalysts were analyzed using X‐ray photoelectron spectroscopy (XPS), confirming the presence of W, V, S, and O elements. Two characteristic peaks in the high‐resolution V 2*p* spectrum of W‐V_3_S_4_ were observed at 514.33 and 516.89 eV, corresponding to V^2+^ and V^3+^ species, respectively (**Figure** **2**a). Notably, the binding energy of W‐V_3_S_4_ experienced a slight positive shift (0.27 eV) compared to pristine V_3_S_4_, indicating modulation of the electronic structure of V_3_S_4_ after the anchoring of W SAs and resulting in a more partially positive charge (δ^+^).^[^ ^7^ ^]^ On the contrary, the S 2*p* peaks of W‐V_3_S_4_ shifted toward lower binding energy (0.25 eV), indicating electron redistribution in the V_3_S_4_ substrate upon W introduction. This facilitated the adsorption and dissociation of intermediates, thereby improving the intrinsic catalytic activity of W‐V_3_S_4_ by reducing the energy barrier of HER (Figure 2b).^[^ ^21^ ^]^ In Figure S8 (Supporting Information), three prominent peaks appeared in the high‐resolution O 1*s* spectra at 530.11, 530.9, and 532.45 eV, indicating the existence of metal‐oxygen (M─O) bonds, metal‐hydroxide (M─OH) bonds, and adsorbed water (H_2_O). The high‐resolution W 4f spectra of W‐V_3_S_4_ (Figure 2c) correspond to 35.54, 37.69, and 41.73 eV for the W^4+^ 4*f*_7/2_, 4*f*_5/2_, and W^6+^ 5*p* states, respectively. The successful doping of W SAs into V_3_S_4_ was further verified in agreement with the HADDF‐STEM and XRD spectroscopy results.

X‐ray absorption spectroscopy (XAS) was utilized to investigate the atomic‐level electronic structure and coordination environment of W‐V_3_S_4_. A comparison with W foil, WO_2_, and WO_3_ revealed that the W L_3_‐edge X‐ray absorption near‐edge structure (XANES) (Figure 2d) spectra of W‐V_3_S_4_ (10 202 eV) indicated a positively charged W state between W^4+^ and W^6+^, consistent with XPS analysis. The pre‐edge white line intensity of the W‐V_3_S_4_, showing a strong single peak at ≈10 210 eV, was slightly higher than that of the WO_2_ curve and indicated a very localized W 5*d* state. Employing the Fourier transform extends the X‐ray absorption fine structure (FT‐EXAFS) spectrum, and the wavelet transform (WT) enables high‐resolution analysis in both k and R spaces. The W‐V_3_S_4_ catalyst exhibited two notable coordination peaks at 1.63 and 2.73 Å (phase uncorrected distance), attributable to the first coordination shell of W─O and W─V (Figure 2e,g; Figure S9, Supporting Information). The presence of W─O bonds signified oxidation on the surface of the catalyst, while the absence of the W─W (2.64 Å) bonds suggested the formation of atomically dispersed W SAs, which was consistent with the results of STEM observations. The calculated 1^st^ shell coordinative number (CN) of W─O, derived from the correlation fitting of the W L_3_‐edge EXAFS curves for W‐V_3_S_4_ was 3.16 (Table S3, Supporting Information), indicating that the central W atom was coordinated with three oxygen atoms, which aligned with the XPS analysis.^[^ ^22^ ^]^ As evidenced, the CN value of W─V is only 2.2, suggesting successful anchoring of W SAs into the S_V_ and coordination with two V atoms (Figure 2f). Impressively, the combination of TEM, XPS, and XAS analysis demonstrated that the obtained W‐V_3_S_4_ featured single‐atom W metal centers and specific unsaturated sites, rather than W‐atom aggregation, thereby effectively enhancing the intrinsic activity.^[^ ^1^, ^19^, ^20^, ^23^ ^]^

### 2.2 HER Performance of W‐V_3_S_4_

**Figure 3:**
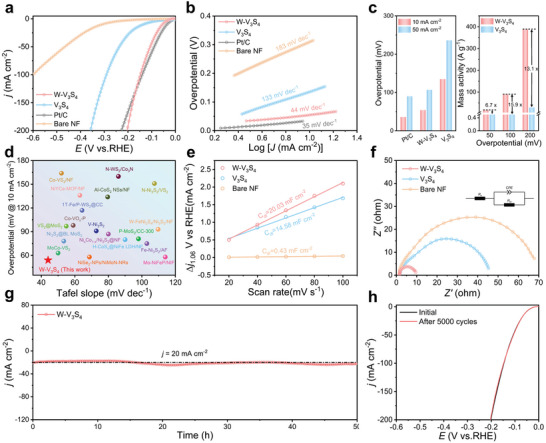
The HER activity evaluation consists of (a) Polarization curves of W‐V_3_S_4_, V_3_S_4_, bare NF, and Pt/C electrodes. b) Tafel plots of W‐V_3_S_4_, V_3_S_4_, bare NF, and Pt/C. c) The overpotentials of various electrodes were assessed at their respective current densities; mass activity for W‐V_3_S_4_ and V_3_S_4_ at overpotentials of 50, 100, and 200mV. d) A comparison of the HER activities among different non‐noble metal catalysts in alkaline solutions. e) *C*_dl_ values. f) EIS Nyquist plots. g) Chronoamperometric curve of W‐V_3_S_4_ electrode at constant potentials for 50 h. h) Comparison of the LSV polarization curves of W‐V_3_S_4_ before and after 5000 CV cycles. All the measurements were 90% iR‐compensated.

Drawing upon the morphological and structural characteristics mentioned earlier, we assessed the HER performance of W‐V_3_S_4_, pristine V_3_S_4_, bare NF, and commercial 20 wt.% Pt/C through linear sweep voltammetry (LSV) curves in a three‐electrode system with a 1 m KOH electrolyte (Figure S10, Supporting Information). With an overpotential of only 54 mV at a current density of 10 mA cm^−2^, W‐V_3_S_4_ demonstrated excellent electrocatalytic activity, as shown in **Figure** **3**a, significantly surpassing V_3_S_4_ (135 mV) and bare NF (309 mV). Notably, the remarkable enhancement in HER activity of W‐V_3_S_4_ could be attributed to the anchoring of W SAs on V_3_S_4_ nanosheets.^[^ ^24^ ^]^ At current densities of 50 mA cm^−2^, as depicted in Figure 3c, W‐V_3_S_4_ exhibited a significantly lower overpotential of 107 mV, demonstrating superior performance compared to V_3_S_4_ (236 mV). Remarkably, the mass activity of W‐V_3_S_4_ reached 88.8 A g_w_^−1^, which was 15.9 times higher than that of V_3_S_4_ (5.59 A g_w_^−1^) at an overpotential of 100 mV. In Figure 3b, the Tafel plots were utilized to evaluate the reaction kinetics. Notably, W‐V_3_S_4_ demonstrated an optimal value of 44 mV dec^−1^, similar to that of commercial Pt/C (35 mV dec^−1^) but significantly lower than that of V_3_S_4_ (133 mV dec^−1^) and bare NF (183 mV dec^−1^). After modification with W SAs, the Tafel slope decreased from 133 to 44 mV dec^−1^, demonstrating the dominance of the Volmer‐Heyrovsky process. The successful anchoring of W SAs facilitated HER kinetics and electron transfer rates. This indicated that the active site for HER was W atoms rather than V atoms in W‐V_3_S_4_. Additionally, the LSV curves of W‐V_3_S_4_ were tested in both acidic (0.5 m H_2_SO_4_) and neutral (1 m PBS) electrolytes (Figure S11, Supporting Information), and the catalytic performances were compared across different environments (Figure S12, Supporting Information). The results demonstrated that W‐V_3_S_4_ exhibited excellent catalytic activity in alkaline, neutral, and acidic conditions, highlighting its broad applicability. Notably, the electrocatalytic activity of W‐V_3_S_4_, as demonstrated by the overpotentials and Tafel slopes, surpassed that of the most recently reported catalysts, as summarized in Figures 3d and Table S4 (Supporting Information).

As depicted in Figure 3e and Figure S13 (Supporting Information), the intrinsic activity of the catalyst was evaluated through electrochemical double‐layer capacitance (*C*_dl_) and the electrochemical active area (ECSA). W‐V_3_S_4_ exhibited a higher *C*_dl_ of 20.02 mF cm^−2^ in the non‐Faradaic region across various scan rates compared to pristine V_3_S_4_ (14.58 mF cm^−2^) and bare NF (0.43 mF cm^−2^). The ECSA values are found to be 500.75, 364.5, and 10.75 cm^2^ for W‐V_3_S_4_, V_3_S_4_, and bare NF in HER, and the normalized polarization curves by ECSA reconfirm the crucial role of W SA anchoring in inducing enhanced HER activity (Figure S14, Supporting Information). This observation suggested a greater availability of active sites, which facilitated the adsorption/desorption of intermediates and favored the catalytic reaction kinetics. Additionally, the turnover frequency (TOF) (Figure S15, Supporting Information) calculated at 200 mV overpotential was 0.37 s^−1^ and 0.055 s^−1^, respectively; this high TOF was a favorable factor for the W‐V_3_S_4_ catalyst to acquire superior intrinsic activity due to the controlled electrode kinetics. The charge transfer resistance (*R*_ct_) was measured by electrochemical impedance spectroscopy (EIS), and the smallest radius corresponded to the higher electronic conductivity. As shown in Figure 3f, the *R*_ct_ of W‐V_3_S_4_ was ≈9.5 Ω, significantly lower than that of V_3_S_4_ (45.2 Ω) and bare NF (67.7 Ω), further suggesting the fastest reaction kinetics rate, and also suggests that single‐atoms anchoring facilitates the separation and transfer of carriers. Moreover, the experimentally measured H₂ production closely matched the theoretical values, demonstrating a Faradaic efficiency of ≈99.5% during the HER process (Figure S16 and Table S5, Supporting Information).

The stability of W‐V_3_S_4_ was evaluated using cyclic voltammetry (CV) and chronoamperometry (CA). After 50 h of continuous reaction at a current density of 20 mA cm^−2^ (Figure 3g), the sustained activity without degradation implied excellent catalytic stability, which is slightly related to the strong interaction between the metal and the carrier. The catalyst demonstrated negligible decay in activity, even when operated continuously at a high current density (200 mA cm⁻^2^) for 100 h (Figure S17, Supporting Information). This conclusion was further confirmed by ICP‐OES tests (Table S2, Supporting Information). Furthermore, the negligible negative shifts were also evidenced by the excellent durability of the LSV curves of W‐V_3_S_4_ before and after 5000 CV cycles (Figure 3h), which profited from the strong interaction between W SAs and V_3_S_4_ nanosheets.^[^ ^25^ ^]^ During the HER process, XRD, XPS data, TEM, and EDS mapping were performed to thoroughly assess the electrocatalyst's durability. For W‐V_3_S_4_, no notable alterations of the XRD diffraction peaks showed a stable crystal structure (Figure S18, Supporting Information). In contrast, the negligible shifts of W 4*f* and V 2*p* XPS peaks suggested unchanged elemental chemical valences and partial surface oxidation observed in the slight positive shifts of S 2*p* and O 1*s* XPS peaks (Figure S19, Supporting Information). Meanwhile, after prolonged operation at the current density of 20 mA cm^−2^, the uniformly distributed W, V, and S elements throughout the V_3_S_4_ nanosheets and the abundant well‐separated bright spots of W SAs remained loaded on the surface and in the S_V_ sites (HAADF‐STEM and the EDS images), determining its non‐agglomerated confinement and particular long‐term stability again (Figures S20 and S21, Supporting Information). These results verified the enduring stability of the structure and morphology of W‐V_3_S_4_ during the long‐term test viability of the HER process. The constructed suitable matrices with exploited defects to immobilize single atoms were essential for the development of functional catalysts for HER. Therefore, this synthetic strategy provides a most promising catalyst as an alternative to commercial precious metal‐based catalysts.

### 2.3 Theoretical Calculation Analysis of W‐V_3_S_4_

**Figure 4:**
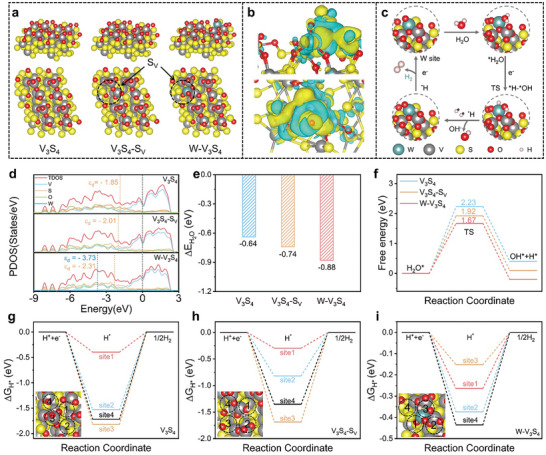
Mechanism and theoretical analysis. a) Theoretical model of the V_3_S_4_, V_3_S_4_‐S_V_, and W‐V_3_S_4_ after optimization. b) Charge density differential image of W‐V_3_S_4_. c)Typical HER mechanism of the W‐V_3_S_4_. d) Electronic density of states (DOS) of three systems. e) Calculated H_2_O adsorption energy of V_3_S_4_, V_3_S_4_‐S_V_, W‐V_3_S_4_. f)Water‐dissociation pathways of V_3_S_4_, V_3_S_4_‐S_V_, W‐V_3_S_4_. g–i) Calculated free energy diagram of the V_3_S_4_, V_3_S_4_‐S_V_, and W‐V_3_S_4_ catalysts for the HER pathway, respectively.

Comprehensive insights into the HER activity of the obtained W‐V_3_S_4_ catalyst and the density functional theory (DFT) calculations were operated to understand the internal electric properties and physical behind (the Supporting Information presented a thorough overview of the calculation procedure for reference).^[^ ^26^ ^]^ Combined with the above analysis, the optimized and relaxed structural models of V_3_S_4_, V_3_S_4_‐S_V_, and W‐V_3_S_4_ with sectional surface oxidation were constructed in **Figure** **4**a. The differential charge density of W‐V_3_S_4_ revealed the existence of charge departure and redistribution (the yellow and green regions denoted areas of charge accumulation and depletion, respectively). In contrast, W atoms exhibited strong chemical bonds with the V_3_S_4_ supports (Figure 4b), further confirming the formation of MSIs and effectively stabilizing the active centers, consistent with XANES and XPS analysis results.^[^ ^27^ ^]^ Figure 4c shows the basic Volmer‐Heyrovsky HER catalytic mechanism diagram of W‐V_3_S_4_, which could be divided into four steps: H_2_O adsorption, H_2_O dissociation, OH desorption, and H reduction.^[^ ^28^ ^]^

The projected density of states (PDOS) analysis for V_3_S_4_, V_3_S_4_‐S_V_, and W‐V_3_S_4_ are shown in Figure 4d. The W‐V_3_S_4_ catalyst possessed the largest DOS value near the Fermi level, resulting in a larger carrier density and improved intrinsic conductivity. For the W‐V_3_S_4_ catalyst, the *d*‐band centers, regarded as an effective descriptor of W and V sites, were −3.73 and −2.31 eV, respectively, which were located further from the Fermi level compared to the V sites in V_3_S_4_ (−1.85 eV) and V_3_S_4_‐S_V_ (−2.01 eV). This indicated weaker intermediate adsorption energy for generating H_2_ on the catalyst surface. Therefore, the W‐V_3_S_4_ catalyst exhibited better HER activity, with the local electronic structure of V_3_S_4_ effectively regulated via the incorporating W SAs and optimized the adsorption/desorption of hydrogen intermediates.^[^ ^29^ ^]^ Additionally, synergistically balancing the adsorption and dissociation of H_2_O molecules on the catalyst surface were typical processes in alkaline HER. The constructed optimization models for the adsorption energy of H_2_O molecules (Δ*E*_H2O_) on the surfaces of V_3_S_4_ (four sites), V_3_S_4_‐S_V_ (four sites), and W‐V_3_S_4_ (four sites) catalysts are shown in Figures S22 and S24 and S26 (Supporting Information), and the Δ*E*_H2O_ values for different sites were calculated in Figure S28 (Supporting Information). As shown in Figure 4e,f, the Δ*E*_H2O_ value (−0.88 eV) of W‐V_3_S_4_ was lower than that of V_3_S_4_ (−0.64 eV) and V_3_S_4_‐S_V_ (−0.74 eV). The energy barrier of the W‐V_3_S_4_ step was as low as 1.67 eV in the water dissociation simultaneously, which was much smaller than that of V_3_S_4_ (2.23 eV) and V_3_S_4_‐S_V_ (1.92 eV), promoting the water dissociation on the surface of W‐V_3_S_4_. These results verified the significant acceleration of the Heyrovsky step and the modulated active sites performing their respective roles in the adsorption/dissociation reaction of H_2_O molecules.^[^ ^30^ ^]^

The Gibbs free energy of hydrogen adsorption (Δ*G*_H*_) stood as another crucial descriptor for evaluating the HER activity, the optimization models for Δ*G*_H*_ of V_3_S_4_, V_3_S_4_‐S_V_, and W‐V_3_S_4_ were shown in Figures S23 and S25 and S27 (Supporting Information). The calculated Δ*G*_H*_ values were presented in Figure 4g–i and Table S6 (Supporting Information), the Δ*G*_H*_ value of W‐V_3_S_4_ at site 3 (−0.152 eV) was closest to the thermoneutral state compared to the value for V_3_S_4_ (−0.395 eV) and V_3_S_4_‐S_V_ (−0.297 eV), implying a less negative d‐band center and more favorable adsorption/desorption for the H* intermedium to generate H_2_ molecule.^[^ ^31^ ^]^ Hence, according to the experiments and theoretical calculations, anchoring W SAs effectively led to charge density redistribution and enhanced the adsorption/desorption of the intermediates, ultimately improving the catalytic activity for the HER process.^[^ ^23b^ ^]^

## 3 Discussion

In summary, we successfully immobilize W single atoms into ultrathin V_3_S_4_ nanosheets (W‐V_3_S_4_) with a high loading of 25.1 wt.% via a sulfur vacancy defect trapping strategy. This strategy exhibits outstanding electrocatalytic activity as well as robust stability, indicating a significant advancement in the development of single‐atom electrocatalysts. The remarkable HER performance comes primarily from 1) the high loading and dispersion of W SAs as active sites, 2) the stronger interaction between S‐rich vacancy defects and W SAs, and 3) the anchoring of W SAs modulating the local electronic structure of V_3_S_4_, effectively accelerating the water dissociation and optimizing the adsorption/desorption of hydrogen intermediates, boosting the whole HER activity. Our research not only demonstrates high‐loading single‐atom catalysts as alternatives to commercial precious metal‐based catalysts, but it also provides a new avenue to extend studies of the heterostructure for hydrogen energy conversion applications.

## 4 Experimental Section

### Synthesis of W‐V_3_S_4_

The W‐V_3_S_4_ catalyst was synthesized by dissolving V_3_S_4_ (100 mg) and WCl_6_ (50 mg) in 40 mL of deionized water and stirring in an ice‐water bath for 12 h. The mixture was washed with ultrapure water and ethanol, centrifuged at 8000 rpm for 5 min, and the black residue was vacuum‐dried overnight at 60 °C. The dried material was ground into powder, transferred to a tube furnace, and heated to 200 °C at a ramp rate of 2 °C min^−1^ under a nitrogen atmosphere for 1 h. After cooling to room temperature, W‐V_3_S_4_ was obtained.

### Synthesis of V_3_S_4_ Nanosheets

V_3_S_4_ nanosheets were synthesized via a hydrothermal method. Ammonium orthovanadate (3 mmol NH_4_VO_3_) and thioacetamide (15 mmol TAA, CH_3_CSNH_2_) were dissolved in 35 mL deionized water with vigorous stirring for 30 min, followed by 15 min of sonication. This solution was transferred to a 50 mL Teflon‐lined autoclave and heated at 180 °C for 24 h. After naturally cooling to room temperature, the black precipitate was repeatedly rinsed with ultrapure water and ethanol and centrifuged at 8000 rpm for 5 min. The precipitate was vacuum‐dried overnight at 60 °C and finely ground with an agate mortar to yield the V_3_S_4_ precursor.

### Electrochemical Characterizations

Electrochemical measurements were performed using a CHI 760E electrochemical workstation in a conventional three‐electrode configuration. The working electrode consisted of W‐V_3_S_4_, while a graphite rod served as the counter electrode, and a Hg/HgO electrode in 1 m KOH acted as the reference electrode. All potentials were referenced to the reversible hydrogen electrode (RHE) using the formula E(RHE) = E(Hg/HgO) + 0.059 × pH + 0.098 V. Polarization curves were recorded in 1 m KOH at a scan rate of 2 mV s^−1^. At the same time, electrochemical impedance spectroscopy (EIS) was conducted over a frequency range from 100 kHz to 0.1 Hz with a 10 mV AC amplitude. All polarization curves underwent a 90% iR correction.

## Conflict of Interest

The authors declare no conflict of interest.

## Supporting information



Supporting Information

## Data Availability

The data that support the findings of this study are available from the corresponding author upon reasonable request.
