# Supplementary material for: Electronic Structure Engineering of Single‐Atom Tungsten on Vacancy‐enriched V3S4 Nanosheets for Efficient Hydrogen Evolution
Source: Adv Sci (Weinh). 2024 Oct 28;12(1):2409855. doi: 10.1002/advs.202409855 (PMC11714179; doi:10.1002/advs.202409855)
Supplement: Supplementary file 1 — Supporting Information [file ADVS-12-2409855-s001.docx]

*Supporting Information*

**Electronic Structure Engineering of Single-Atom Tungsten on Vacancy‐enriched V_3_S_4_ Nanosheets for Efficient Hydrogen Evolution**

Min Xi^a#^, Hua Zhang^a#^, Lingfeng Yang^a^, Youyu Long^a^, Yifan Zhao^a^, Anran Chen^a,b^*, Qiaozhi Xiao^c^, Tingting Liu^a,b^, Xuechun Xiao^a^*, Guangzhi Hu^a, c^*

*^a^* Yunnan Key Laboratory of Electromagnetic Materials and Devices, School of Materials and Energy, Yunnan University, Kunming 650091, China

*^b^* Electron Microscopy Center, Yunnan University, Kunming 650091, China

*^c^* Institute for Ecological Research and Pollution Control of Plateau Lakes, School of Ecology and Environmental Science, Yunnan University, Kunming 650091, China

^#^ Equal author contribution

*Corresponding Authors Email: anran@ynu.edu.cn (A. Chen), [xchxiao@ynu.edu.cn](mailto:xchxiao@ynu.edu.cn) (X. Xiao), guangzhihu@ynu.edu.cn (G. Hu).

Table of Contents

1.Supporting experimental section··················································· ·······3

2.Supporting figures···········································································10

3.Supporting tables············································································38

4. Supporting references······································································44

1. **Supporting experimental section**

**1.1 Materials synthesis**

*Chemicals and materials*

Ammonium orthovanadate (NH_4_VO_3_, ≥99.0%), thioacetamide (TAA, CH_3_CSNH_2_, ≥99.0%), Tungsten (VI) chloride (WCl_6_), potassium hydroxide (KOH) purchased from Aladdin, Pt/C (20wt% Pt on Vulcan XC-72R) and Nafion (5 wt%) were purchased from Alfa Aesar. All chemicals were used as received without further purification, and all aqueous solutions were prepared with ultrapure water (>18.25 MU cm) obtained from a Millipore system.

*Pretreatment of nickel foam*

The nickel foam (2 × 3 cm^2^) was first cleaned with acetone, water, and ethanol by ultrasonic treatment for 10 minutes respectively. After that, the nickel foam was cleaned in a 3 M HCl solution for 15 minutes, followed by sonication sequentially in water and ethanol several times. The nickel foam was taken out and cleaned thoroughly with water and ethanol several times before being vacuum-dried.

*Synthesis of W-V_3_S_4_*

The synthesis of the W-V_3_S_4_ catalyst proceeded as follows: firstly, V_3_S_4_ (100 mg) and WCl_6_ (20 mg) were dissolved in 40 mL of deionized water and stirred in an ice-water bath for 12 h. The resulting mixture was washed with ultrapure water and ethanol, followed by centrifugation at 8000 rpm for 5 minutes to collect the black precipitate, which was then vacuum-dried overnight at 60 °C and ground into power. The powdered material was finely ground using an onyx mortar, transferred into a tube furnace, and heated to 200 °C at a fixed ramp rate (2 °C/min) under a flowing N_2_ atmosphere for 1 h. Upon cooling the tube furnace to room temperature, W-V_3_S_4_ was obtained.

*Synthesis of V_3_S_4_ nanosheets*

V_3_S_4_ nanosheets were synthesized via a hydrothermal approach. In general, 3 mmol ammonium orthovanadate (NH_4_VO_3_) and 15 mmol thioacetamide (TAA, CH_3_CSNH_2_) were dissolved in 35 mL deionized water under vigorous stirring at room temperature for 30 minutes, followed by 15 minutes of sonication. The resulting homogenous solution was then transferred to a 50 mL Teflon-lined autoclave and maintained at 180 °C for 24 h. After natural cooling to room temperature, the black precipitate was subjected to repeated rinsing with ultrapure water and ethanol, followed by centrifugation at 8000 rpm for 5 minutes to collect the precipitate. Subsequently, the precipitate was vacuum-dried overnight at 60 °C. Finally, the obtained black precipitate of V_3_S_4_ was finely ground using an agate mortar to yield the V_3_S_4_ precursor.

*Synthesis of the electrode of Pt/C on nickel foam*

For the synthesis of the Pt/C electrode on nickel foam, 10 mg of commercial Pt/C and 50 μL of Nafion (5 wt%) solution were dissolved in 1 mL of 4:1 v/v water/ethanol through at least 30 min of sonication to form a homogeneous ink. Then, 160 μL of the ink was dropwise loaded onto the nickel foam (1×1 cm^2^). Finally, the electrode was dried at room temperature for 24 h. The loading amount of Pt/C catalysts was estimated to be 1.6 mg cm^−2^.

**1.2 Materials Characterizations:**

Powder X-ray diffraction (XRD) pattern was recorded on a Rigaku TTRⅢ-18KW diffractometer operated at 40 kV voltage and 30 mA current using Cu Kα radiation (λ=1.5418 Å) in the range of 10-90^0^. X-ray photoelectron spectroscopy (XPS) studies were performed using a Thermo Fisher K-Alpha^+^ equipped with monochromatic Al Ka radiation (150 W, 5 kV at 1486.6 eV). The chamber pressure for the spectrometer was kept at 10^−9^ Torr. The surface charge was corrected by referencing the spectra to the C 1*s* peak for the C-C bond at a binding energy of 284.8 eV. Scanning Transmission Electron Microscopy (STEM, Titan Cubed Themis G2 300). The transmission electron microscope (TEM) analysis was performed on a JEOL JEM-2100 transmission electron microscope operated at an accelerating voltage of 200 kV. Samples for TEM analysis were prepared by dropping dilute solutions of nanocrystals onto carbon-coated grids and letting the solvent evaporate. The Raman spectrum was recorded on a HORIBA Raman microscope with a laser wavelength of 532 nm (LabRAM HR Evolution) for surface characterization. The content of metal atoms was detected by an inductively coupled plasma-optical emission spectrometer (ICP-OES). The Sv of the samples was detected by electron paramagnetic resonance (EPR) (Bruker, E500-9.5/12). The Brunauer-Emmett-Teller (BET) surface analyzer (ASAP 2020 Plus system, Micromeritics Instrument Corp, USA) was used to calculate the surface area and pore size via nitrogen adsorption−desorption isotherms.

- 1. **Electrochemical Measurements**

Electrochemical measurements are performed with a CHI760E electrochemical workstation in a standard three-electrode system using W-V_3_S_4_ as the working electrode, a graphite rod as the counter electrode, and a Hg/HgO electrode (in 1 M KOH) as the reference electrode. Typically, 20 mg of W-V_3_S_4_ sample and dispersed in 1000 mL of mixed solution (50 μL Nafion, 250 μL isopropanol, and 700 μL ethanol), sonicated for 30 min, and 50 μL of homogeneous ink droplets were taken on 1×0.5 cm^−2^ nickel foam and dried naturally for 12 h (catalyst loading of 2 mg cm^−2^). All measured potentials were referred to the reversible hydrogen electrode (RHE) using the following equation: E(RHE) = E(Hg/HgO) + 0.059 × pH + 0.098 V, and the current densities (*j*) were normalized by geometric surface area. To make a more reliable comparison, Pt/C was loaded on NF with the same loading as that of W-V_3_S_4_. The polarization curves were recorded in 1 M KOH with a scan rate of 2 mV s^−1^. Electrochemical impedance spectroscopy (EIS) was carried out from 100 kHz to 0.1 Hz at the given potential with an AC amplitude of 10 mV. All polarization curves were 90% iR-corrected.

- 1. **Tafel Slope**

The Tafel slope is an important electrochemical parameter that reflects the sensitivity of the current response to the overpotential. By assuming a specific rate-determining step, the theoretical Tafel slope can be derived based on the microkinetic model, whereby the experimentally determined Tafel slope can be compared with the calculated counterpart to clarify the reaction mechanism and the surface kinetics. The experimentally observed overpotential ƞ (V) at any current is given by the following equation:

ƞ = *a* + *blnj* + *jR* Eq. (1)

where a (V) is the Tafel constant, b (V dec^−1^) is the Tafel slope, j (A cm^−2^) is the current density, and R (Ω) is the total resistance of the system assumed to be constant and independent of the current. Differentiating Eq. (1) with respect to current density gives Eq. (2), from which b and R can be easily obtained by plotting dƞ/dj vs. 1/j.

$\frac{d_{ƞ}}{d_{i}}=\frac{b}{j}+R$ Eq. (2)

Knowledge of R allows correction of the experimental overpotential by subtraction of the ohmic drop, jR, according to the following equation:

$ƞ_{coor}=ƞ-\mathrm{jR}$ Eq. (3)

In the numerical calculation, the derivative dƞ /dj was replaced by Δƞ/Δj calculated from each pair of two consecutive experimental points.

**1.5 Electrochemically active surface area (ECSA) calculation**

The active surface area of each catalyst was estimated from their electrochemical capacitances, which can be measured using a simple cyclic voltammetry method. The current was measured in a narrow potential window without a faradaic process. We sweep the potential between 0.1 to 0.2 V vs RHE (HER) at each of five different scan rates (20, 40, 60, 80, and 100 mV s^−1^). A linear trend is observed by plotting the difference in current density (J) between anodic and cathodic sweeps (ΔJ) at a fixed potential against the scan rate. The fitting slope is twice the double-layer capacitance (*C_dl_*), which is linearly promotional to the ECSA. The specific capacitances (*C_s_*) for a flat surface are normally between 0.02-0.06 mF cm^−2^ (*C*_s_ = 0.040 mF cm^−2^). The calculation formula is as follows:

*ECSA*=$\frac{C_{dl} \left( catalyst \right) mF {cm}^{-2}}{C_{s}\cdot per ECSA {cm}^{-2}}$

These values of *C_dl_* permit comparison of the relative surface activity of different electrodes, particularly in the same electrolyte.

**1.6 Active site density(n) and Turnover frequency (TOF) calculations**

The active site density (n) has been calculated using the following formula (according to the reference: *ACS Nano 2020, 14, 5, 5426–5434; ACS Appl. Nano Mater. 2022, 5, 1, 1385–1396; ACS Nano 2022, 16, 3, 4861–4875*):

n = Q/kF, where k represents the number of electrons involved in the HER process. Q is the Voltammetric charge, and F is the Faraday constant (F) ~ 96485 C/mol.

n = Q/2F where k = 2 in the HER process.

To calculate the Voltammetric charge, the CV analysis is carried out in 1M Phosphate buffer Saline (PBS) by fixing a potential window of −0.2 V to 0.6 V (vs RHE) at a scan rate of 50 mV/s. The active site density of W-V_3_S_4_ is found to be 2.25 × 10^−5^ mol/cm^2^ for HER. Here, we assume that all the active surface sites are involved in HER activity. Using this active site density (n), the TOF is calculated by the following formula:

For HER, TOF = *j*/2nF

where j (A cm^−2^) is the current density measured by the LSV plots in 1 M KOH for HER.

**1.7 X-ray absorption (XAS) characterization**

The X-ray absorption spectroscopy spectra were collected at the BL01C1 Beamline in Shanghai Synchrotron Radiation Facility. The typical energy of the storage ring was 1.5 GeV, and the electron current was 180 mA in the top-up mode. The white light was monochromatized by a Si (111) double-crystal monochromator and calibrated with the corresponding standard metal foil. Samples were pressed into thin slices and positioned at 90° to the incident beam in the sample holder. The XAS spectra were recorded in transmission mode with two ion chambers. All samples were scanned in the range of 10000 to 10500 eV with 0.1 eV steps to measure the L_3_ absorption edge of W. Additionally, W foil, WO_2_, and WO_3_ powder were used as references for W^0^, W^4+^, and W^6+^valence states, respectively. Athena and Artemis software were used to analyze the XANES and EXAFS data, respectively. The coordination number (*N*), bond distance (*R*, in the unit of Å), and Debye-Waller factor (σ^2^) were all extracted from EXAFS data.

To further investigate the first-shell backscattering atoms and detect light and heavy scatters, wavelet transform (WT) analysis was employed using the Igor Pro script developed by Funke et al. (Funke et al., 2005, Funke, H., Scheinost, A.C., Chukalina, M., 2005. Wavelet analysis of extended X-ray absorption fine structure data. *Physical Review B* 71). This qualitative analysis was primarily focused on the nature of the backscattering atoms as well as the bond lengths owing to the fine resolution in both wavenumbers k and radial distribution function R, and complemented the limitation of FT analysis. A Morlet wavelet was chosen as the basis mother wavelet, and the parameters (*η* = 8, *σ* = 1) were used for a better resolution in the wave vector k.

**1.8 Theoretical calculation section**

In this work, all DFT calculations were constructed and implemented in the Vienna ab initio simulation package (VASP).^[27,28]^ The first-principle density functional theory calculations are performed with the projector augmented wave (PAW) method. The generalized gradient approximation of the Perdew-Burke-Ernzerhof (GGA-PBE) function is used to treat the exchange function.^[29-31]^ In our calculation, the V_3_S_4_(310) surfaces have been established. The cut-off energy of the plane-wave basis is set at 550 eV to optimize calculations of atoms and surfaces. Partial occupancies of the Kohn−Sham orbitals were allowed using the Gaussian smearing method and a width of 0.02 eV. The Brillouin zone integration is performed using 3×3×1 Monkhorst and Pack k-point sampling for a structure, and the k-point sampling was obtained from the Monkhorst−Pack scheme mesh for DOS calculation. The self-consistent calculations apply a convergence energy threshold of 10^−5^ eV. The dimer method is used to search for transition states, and the transition state calculations converge with a threshold of 10^−7^ eV.^[32]^ The equilibrium lattice constants are optimized with maximum stress on each atom within 0.02 eV Å^−1^. The vacuum spacing in a direction perpendicular to the plane of the structure is 15 Å. Spin polarizations was considered in all calculations.

**2. Supporting figures**


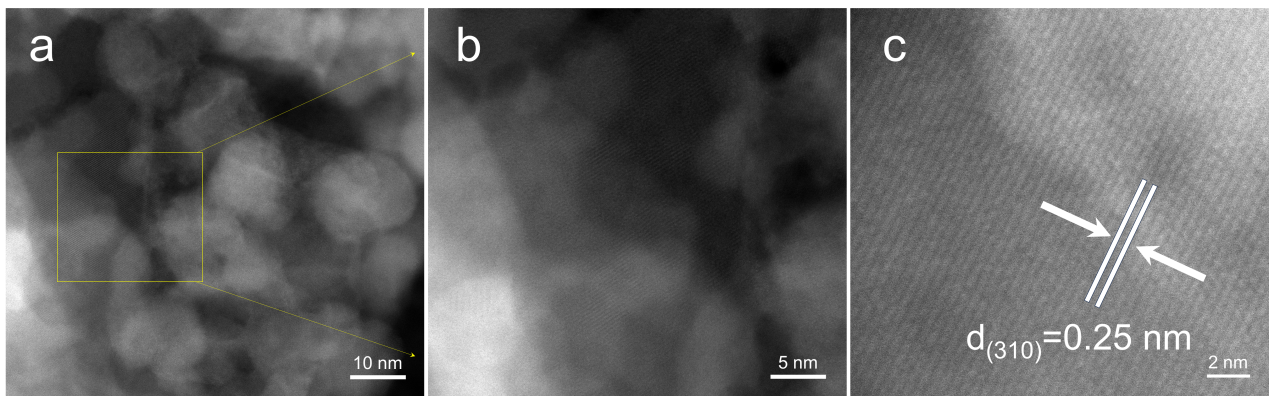


**Figure S1.** (a-c) HRTEM images of V_3_S_4_ nanosheets with different magnifications.


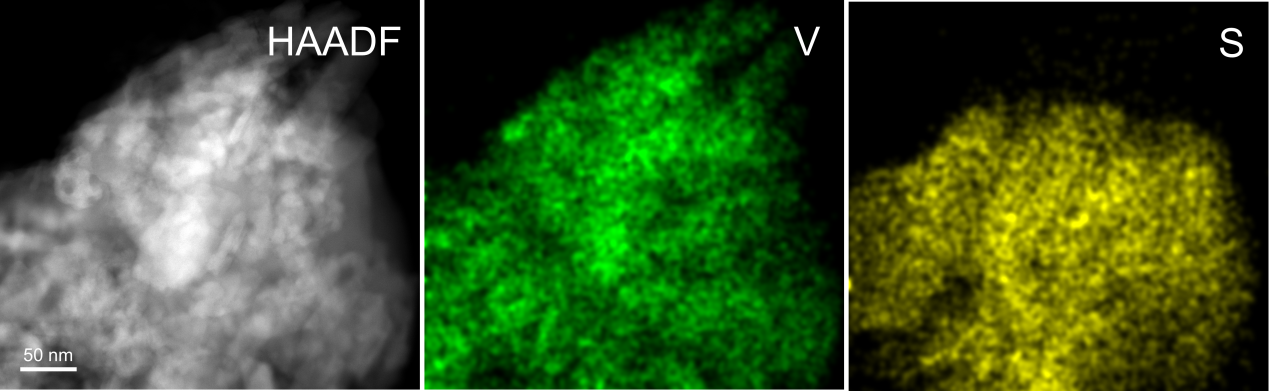


**Figure S2.** HAADF-STEM image and the corresponding EDX elemental mappings in V_3_S_4_.

_
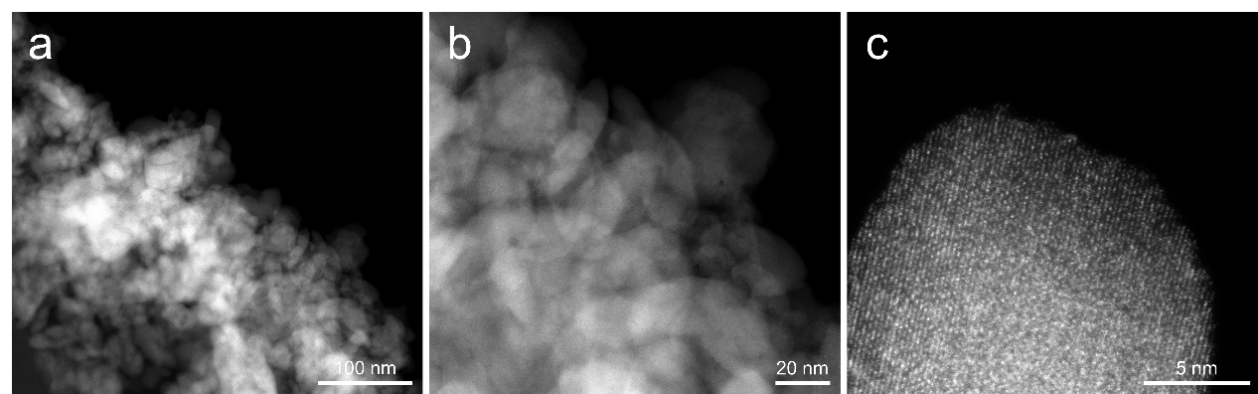
_

**Figure S3.** (a-c) HRTEM images of W-V_3_S_4_ nanosheets with different magnifications.


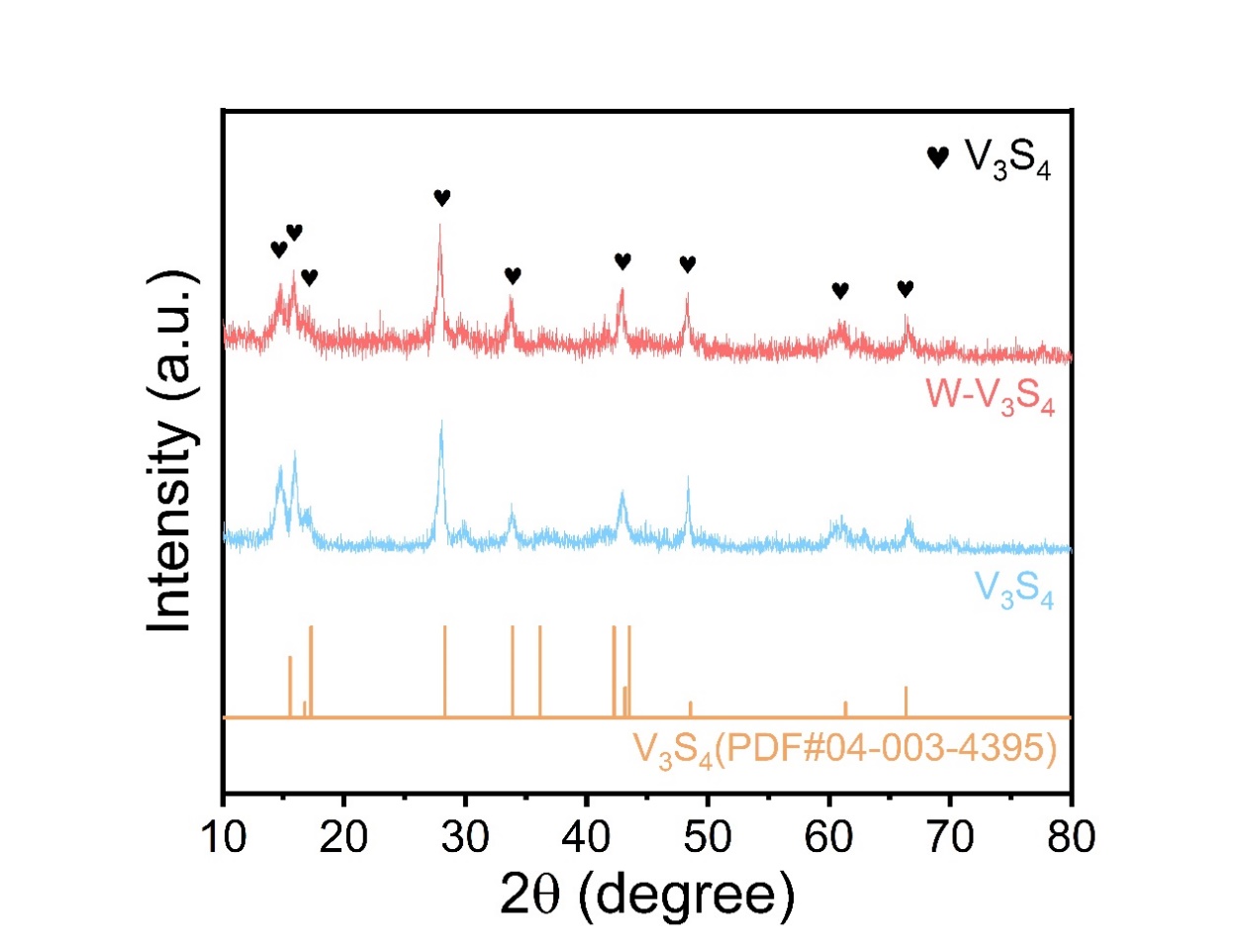


**Figure S4.** XRD patterns of W-V_3_S_4_ and V_3_S_4_


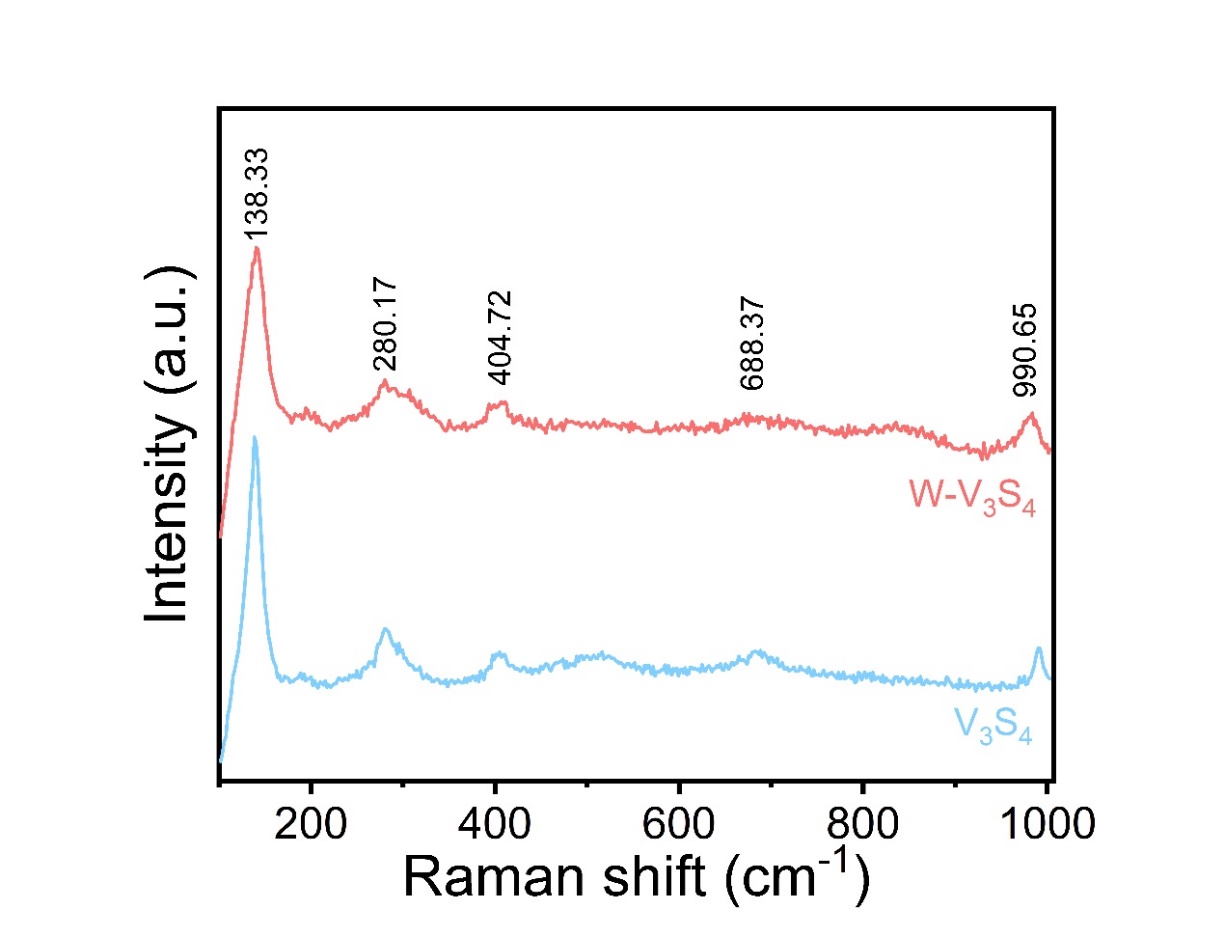


**Figure S5.** Raman spectra of W-V_3_S_4_ and V_3_S_4_


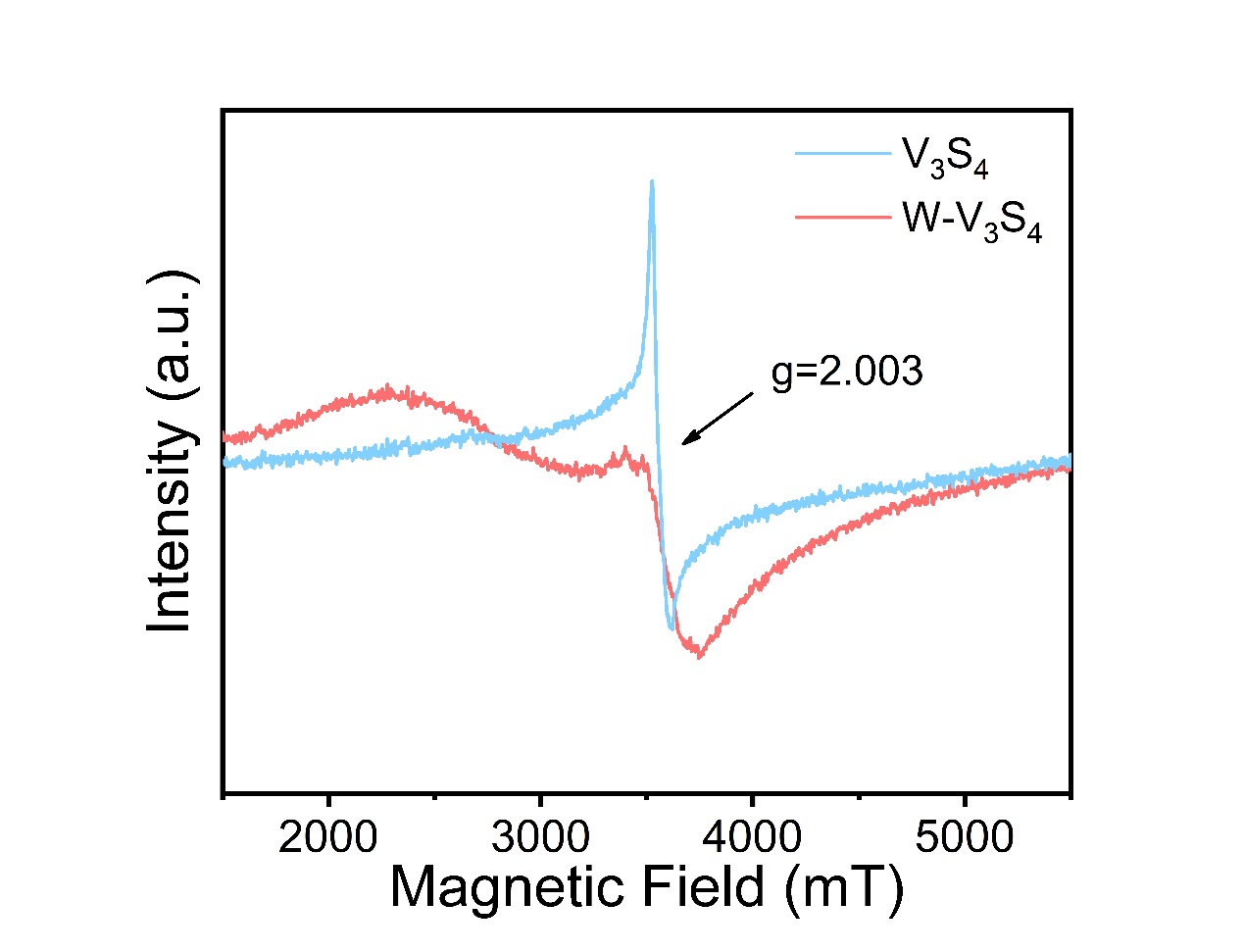


**Figure S6.** EPR spectra of V_3_S_4_ and W-V_3_S_4_


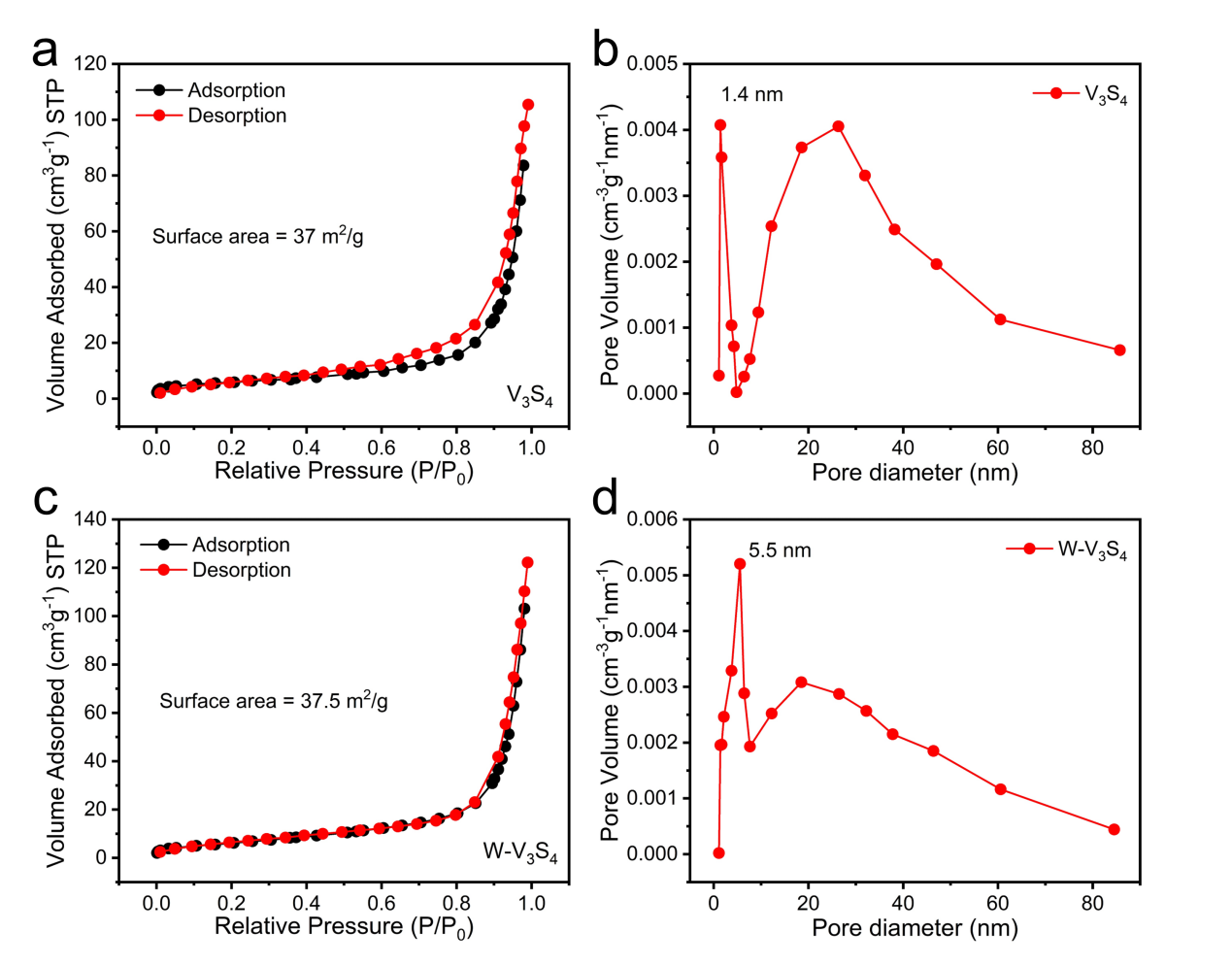


**Figure S7.** (a, c) Nitrogen adsorption-desorption isotherms of V_3_S_4_ and W-V_3_S_4_ and (b, d) corresponding BJH pore size distributions curve of V_3_S_4_ and W-V_3_S_4_ sample.


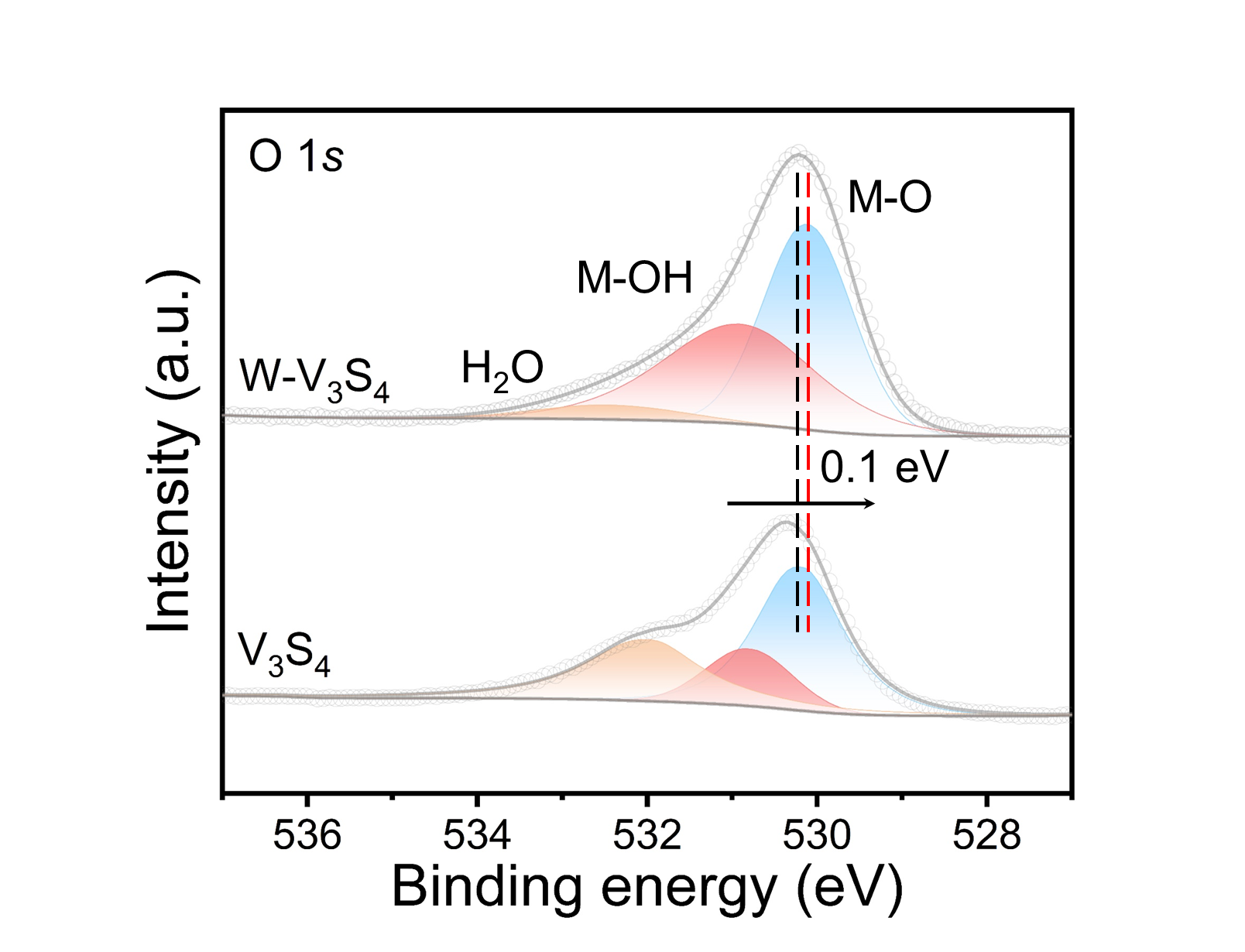


**Figure S8.** High-resolution XPS spectra of O 1s in W-V_3_S_4_ and V_3_S_4_.


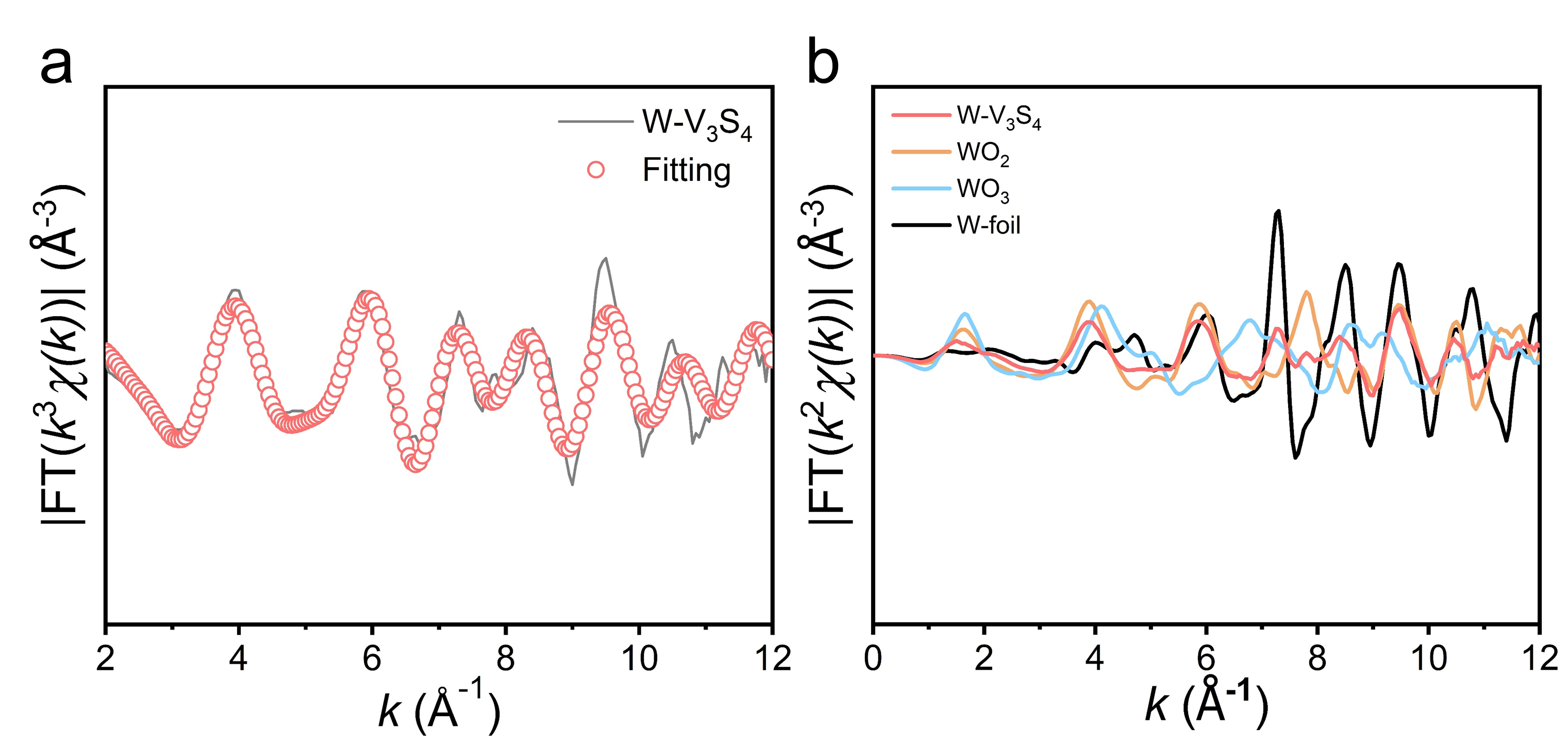


**Figure S9.** (a) *k* space EXAFS spectra with fits for W-V_3_S_4_ and (b) *k* space EXAFS spectra for W-V_3_S_4_, WO_2_, WO_3_, and W-foil.


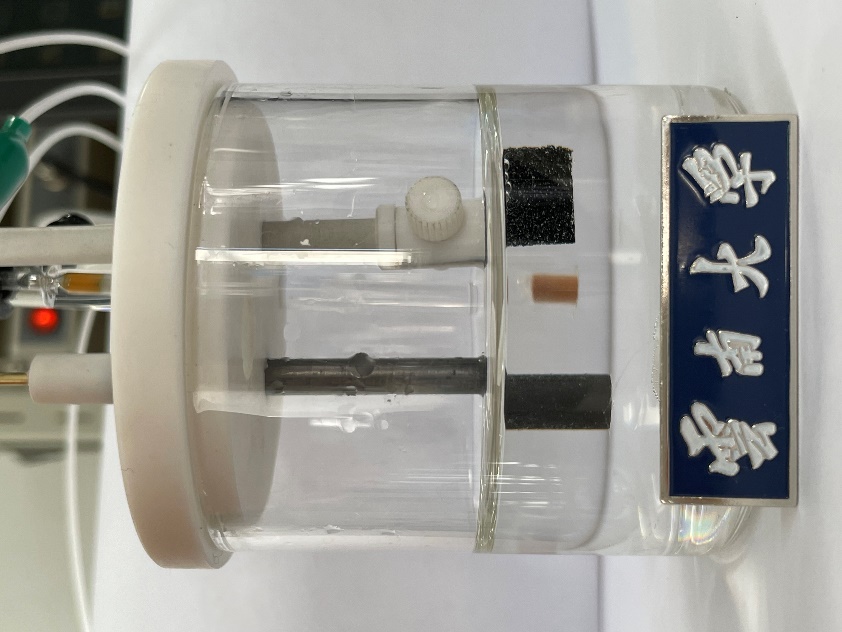


**Figure S10.** Optical photographs of a standard three-electrode system of W-V_3_S_4_ in 1 M KOH electrolyte.

_
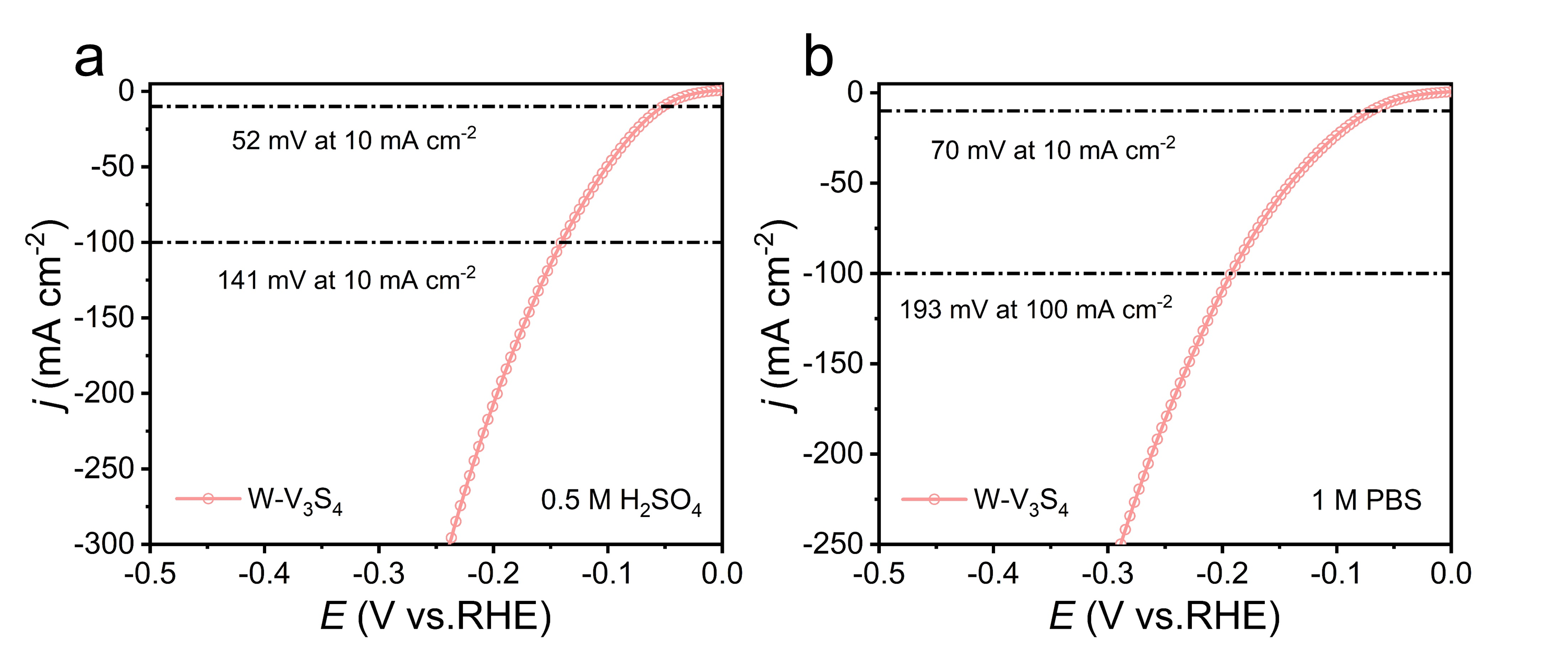
_

**Figure S11.** LSV curves of HER for W-V_3_S_4_ in (a)0.5 M H_2_SO_4_ and (b) 1 M PBS.

_
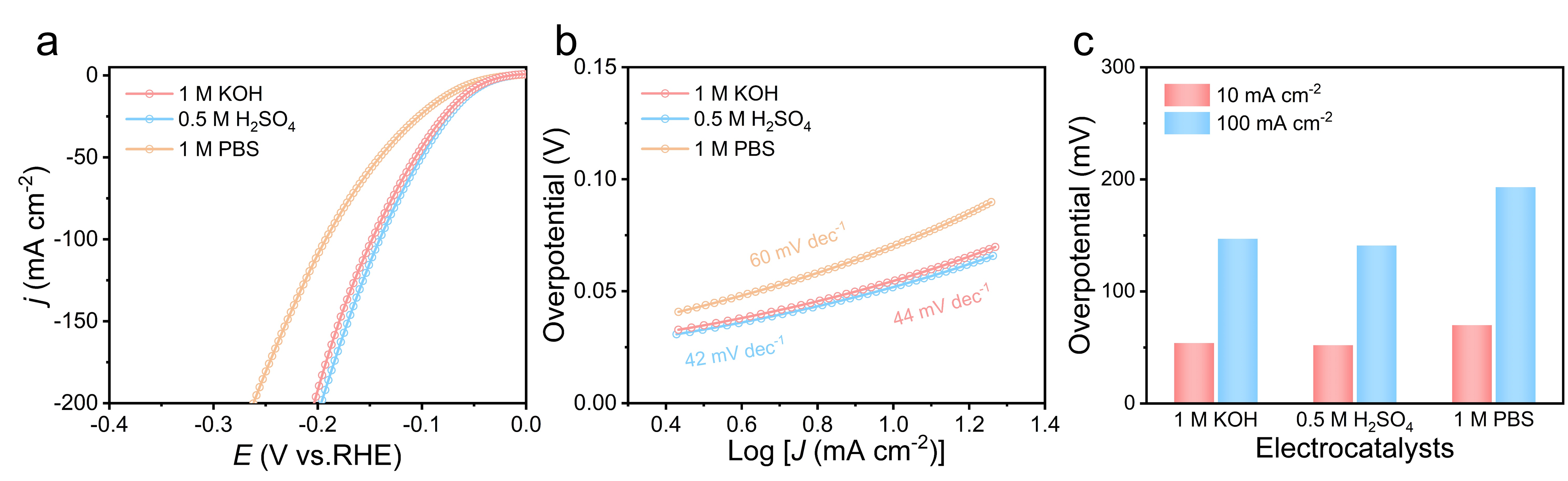
_

**Figure S12.** The HER activity evaluation consists of (a) Polarization curves of W-V_3_S_4_ in 1 M KOH, 0.5 M KOH and 1 M PBS conditions, respectively. (b) Tafel plots. (c) Assessment of overpotentials under various conditions at current densities of 10 mA cm^−2^ and 100 mA cm^−2^.


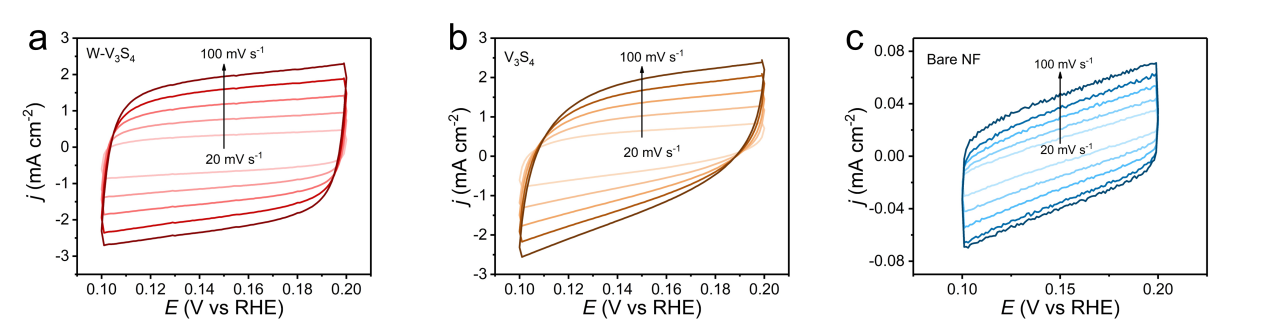


**Figure S13.** In the non-faradic capacitance current range of 20 to 100 mV s^−1^, cyclic voltammograms of (a) W-V_3_S_4_, (b) V_3_S_4_, (c) Bare NF were recorded.


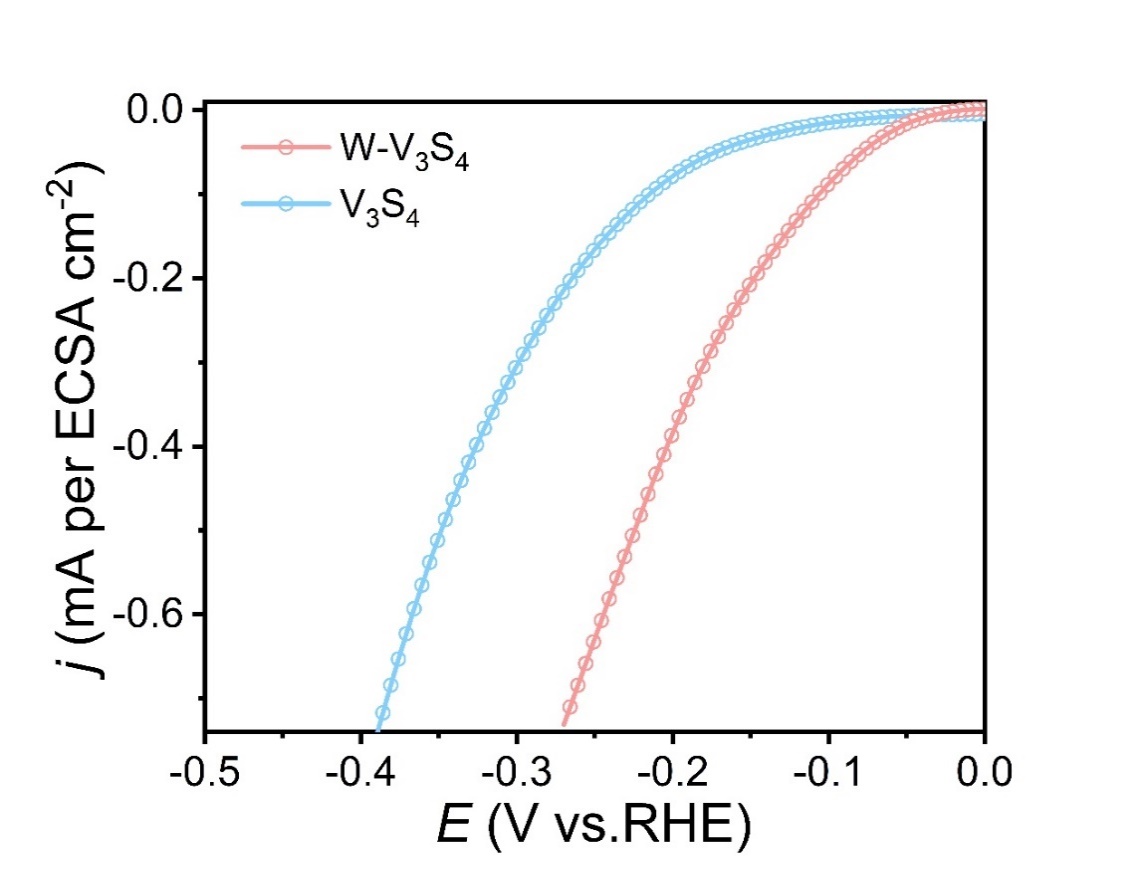


**Figure S14.** ECSA-normalized polarization curves of W-V_3_S_4_ and V_3_S_4._


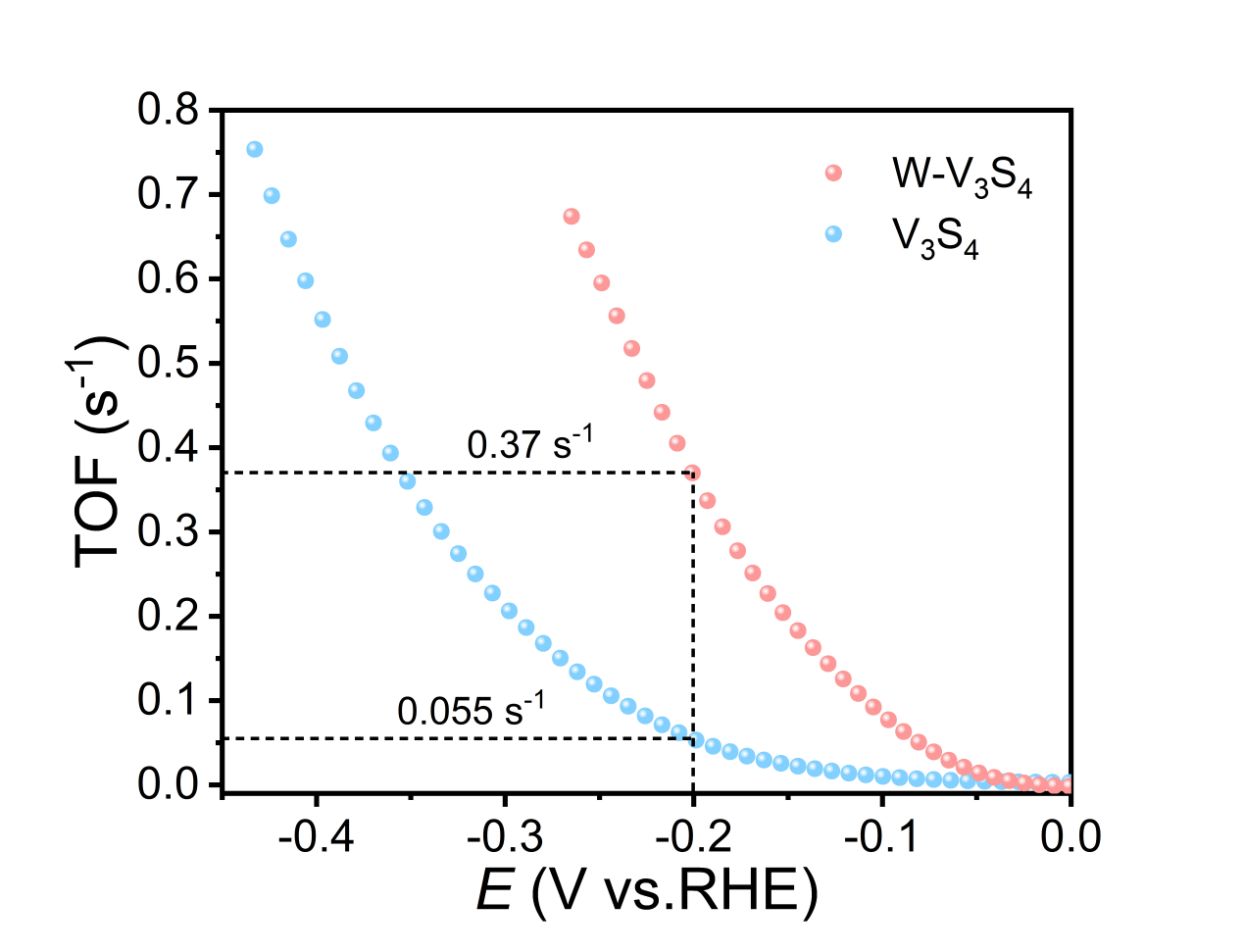


**Figure S15.** The calculated TOF curves of the W-V_3_S_4_ and V_3_S_4_ electrodes for HER.


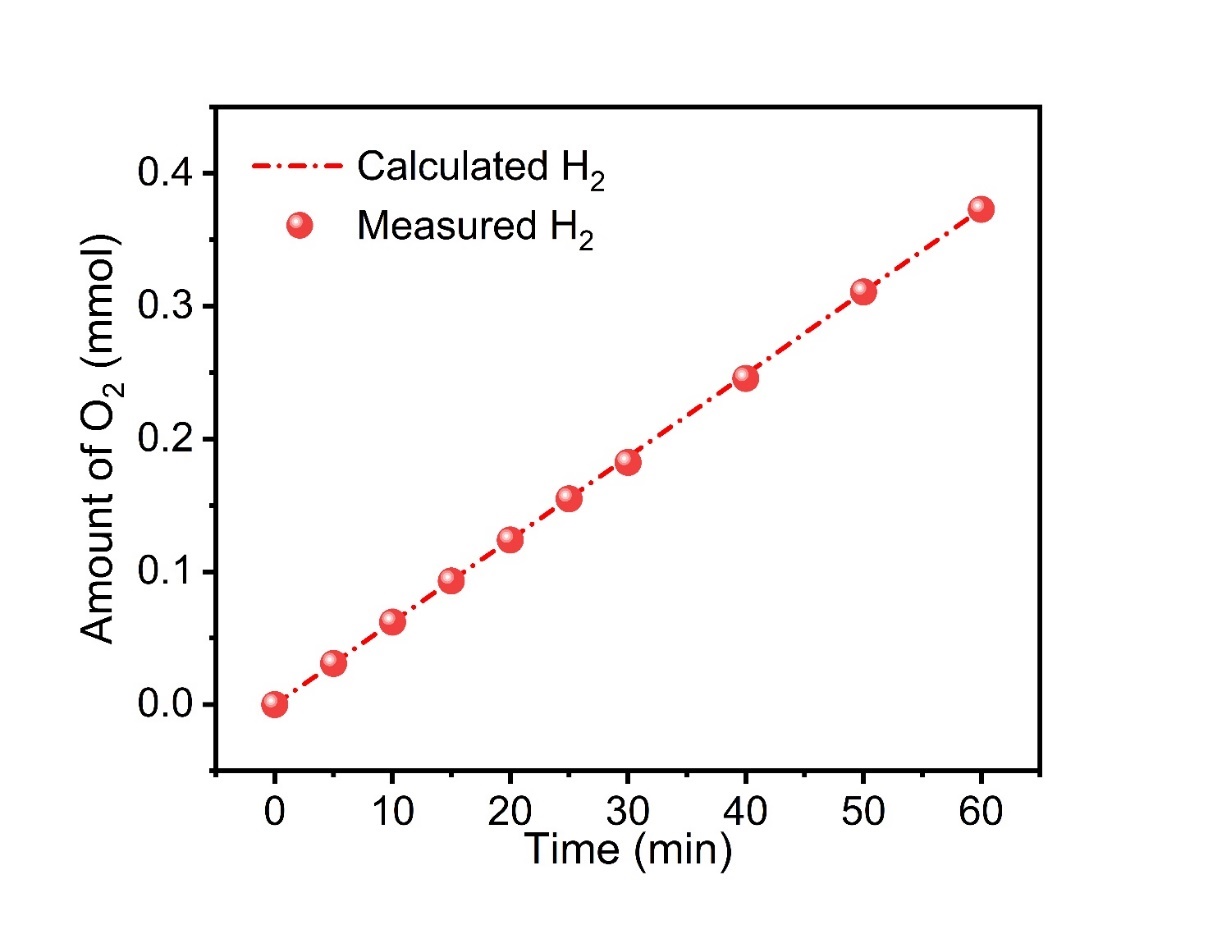


**Figure S16.** Experimentally measured H_2_ generation versus theoretically calculated quantities for W-V_3_S_4_.


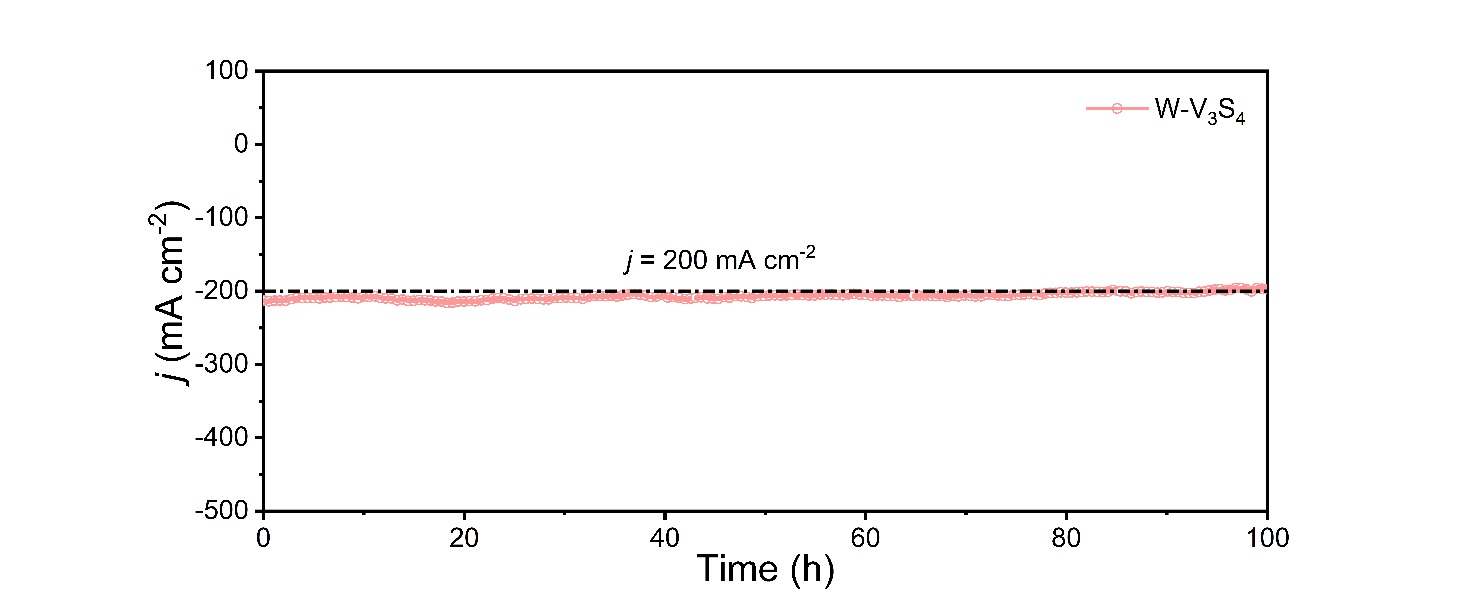


**Figure S17.** Chronoamperometric curve of W-V_3_S_4_ electrode at constant potentials for 100 h.

_
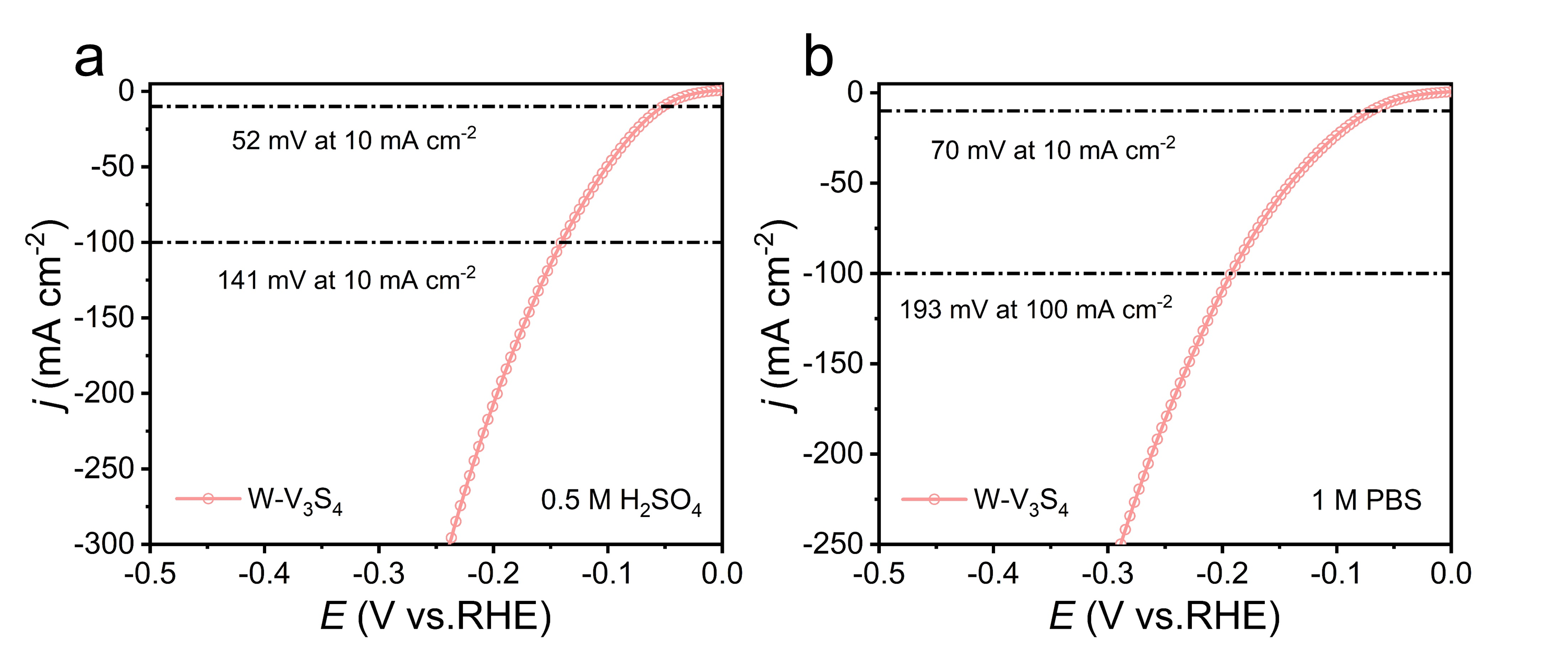

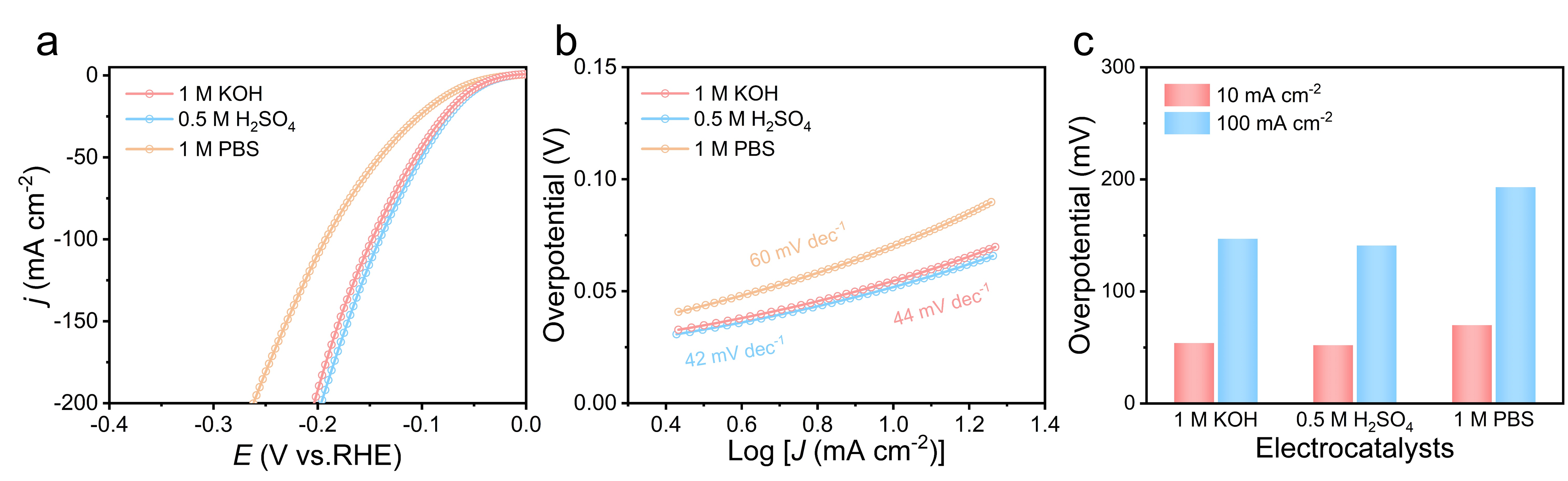
_
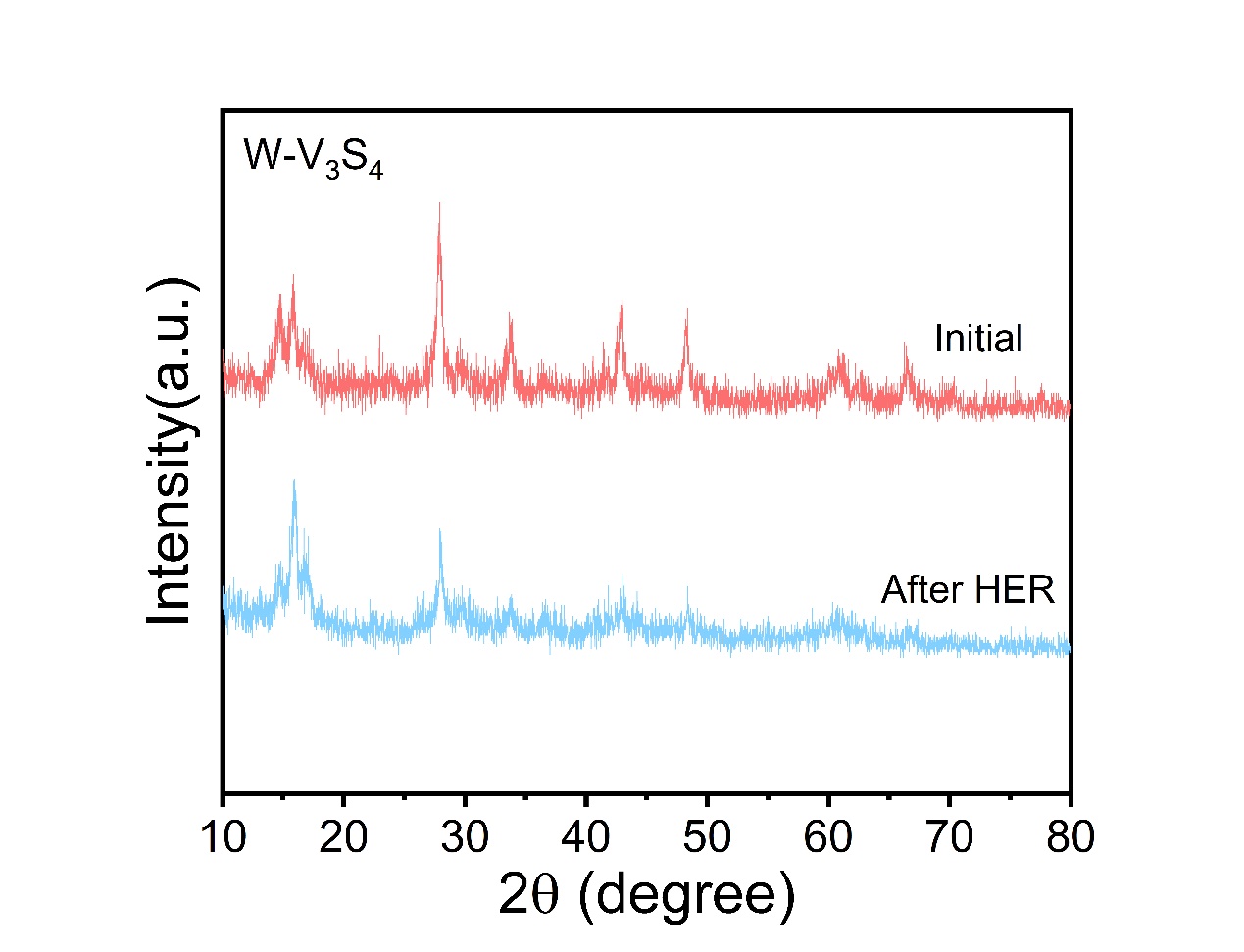


**Figure S18.** XRD pattern of W-V_3_S_4_ after the HER durability test.


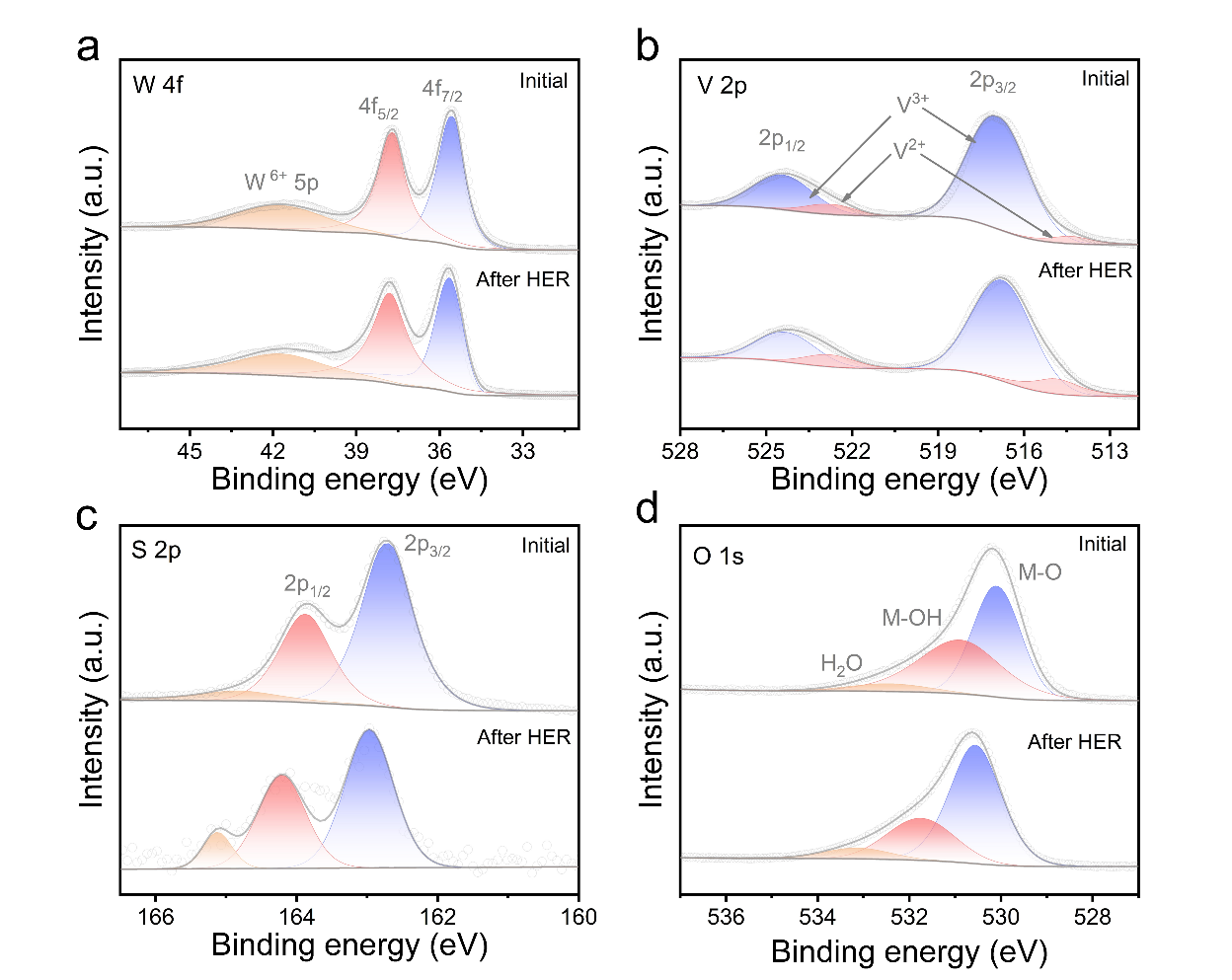


**Figure S19.** Post characterizations of W-V_3_S_4_ catalyst before and after HER stability test. (a) W 4*f*, (b)V 2*p*, (c) S 2*p,* and (d) O 1*s* core level XPS spectra.

**
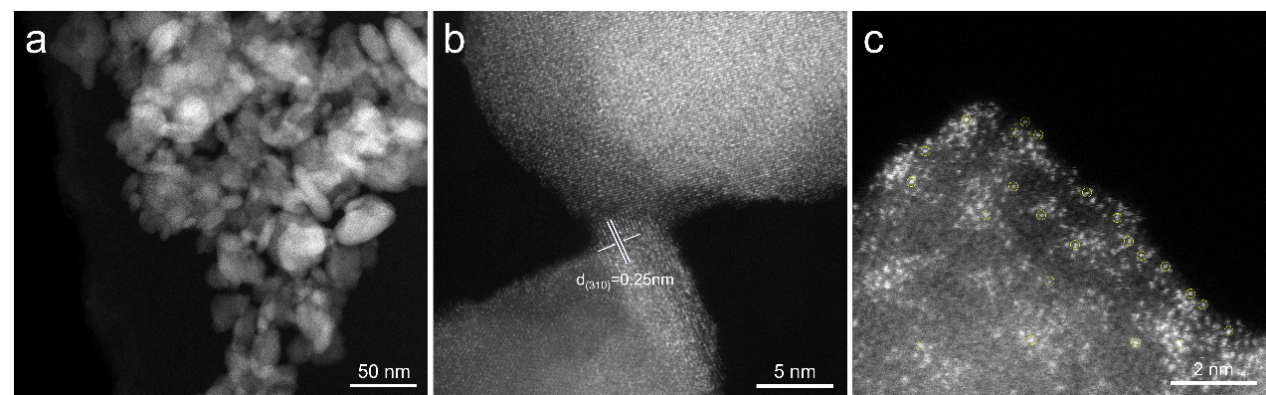
**

**Figure S20.** HRTEM images of W-V_3_S_4_ after the HER durability test at different magnifications.

**
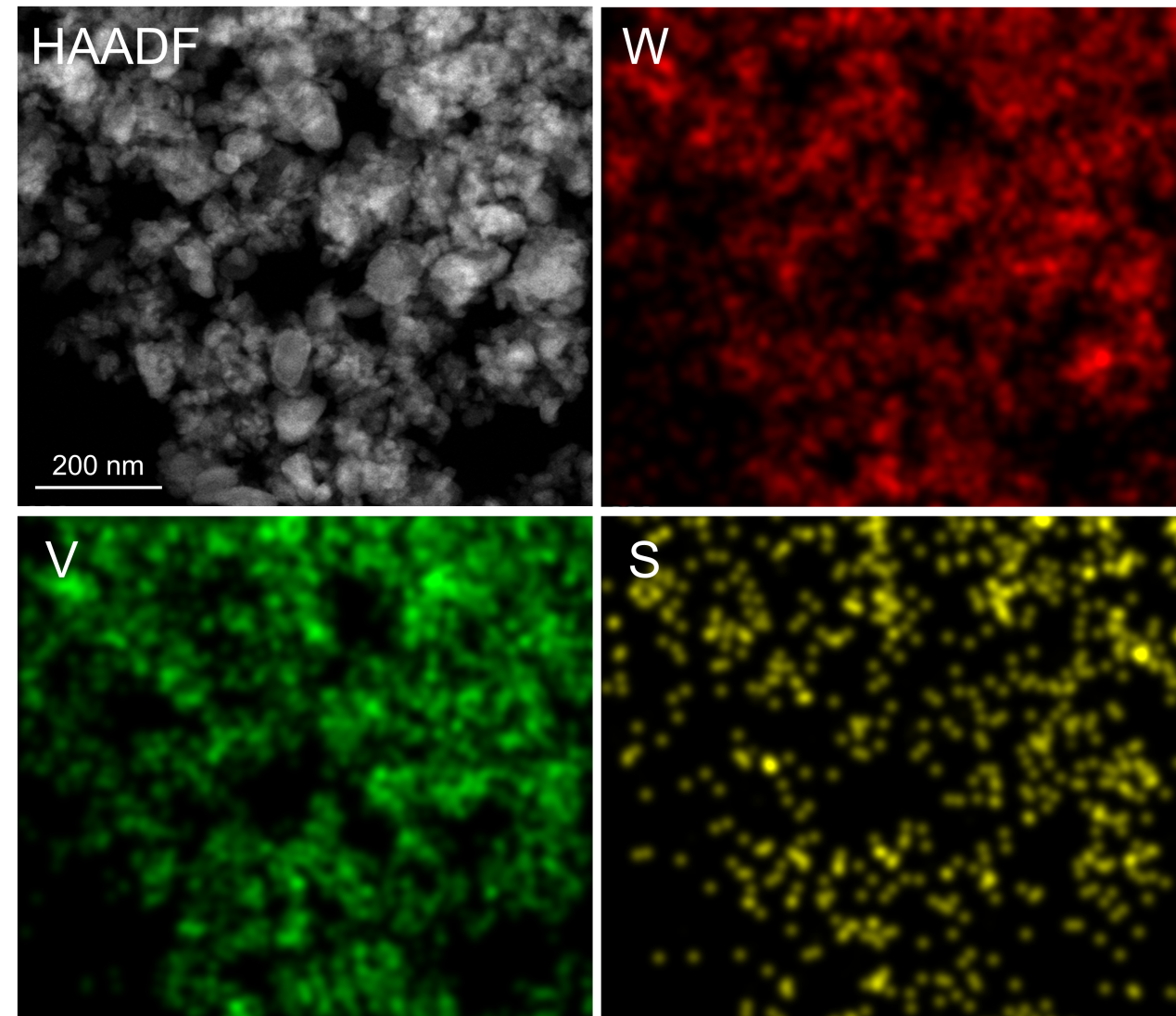
**

**Figure S21.** HAADF-STEM image and the corresponding EDX elemental mapping images for W, V, and S of W-V_3_S_4_ after the HER durability test.


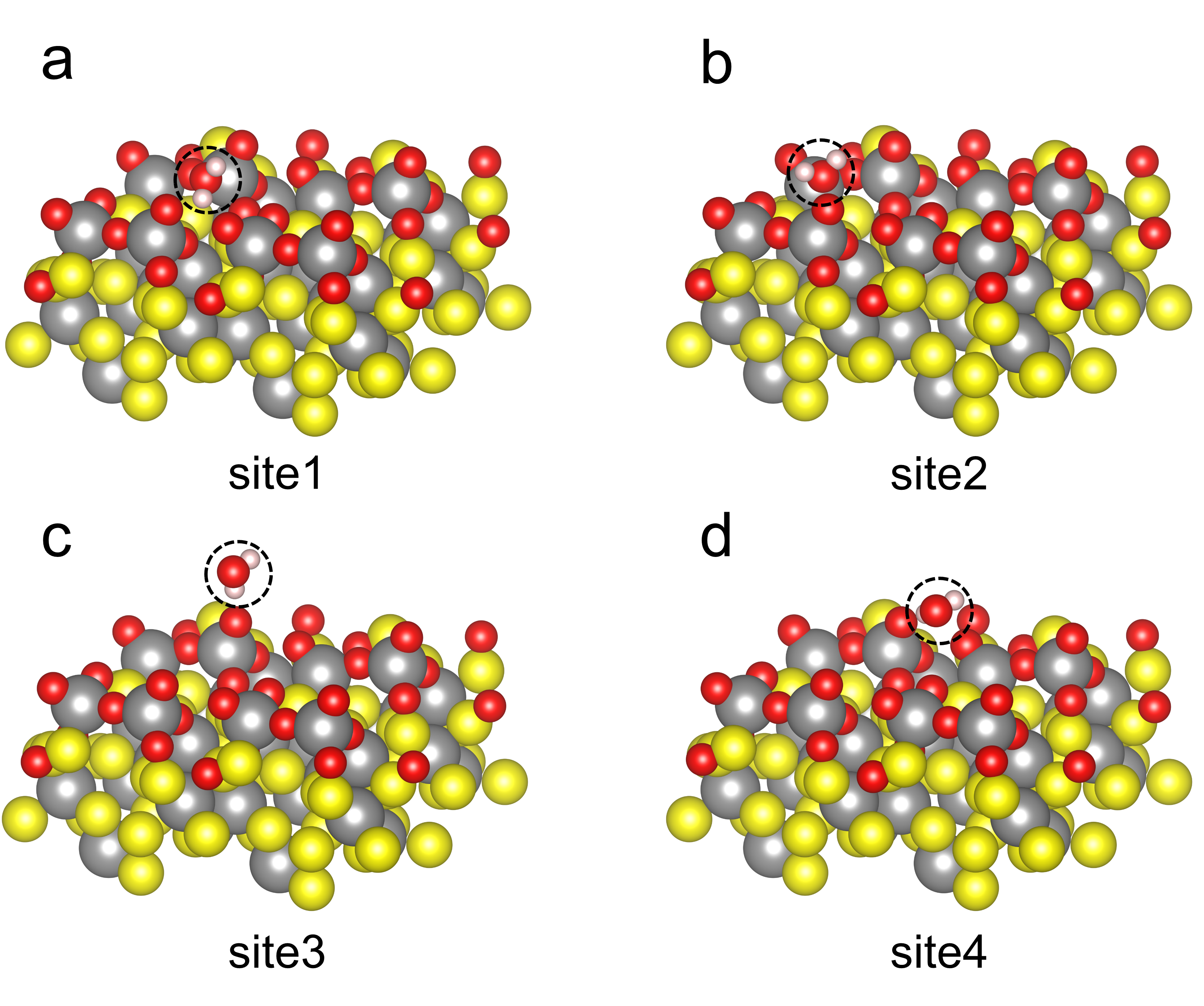


**Figure S22.** Theoretical model of H_2_O adsorption at different sites of V_3_S_4_.


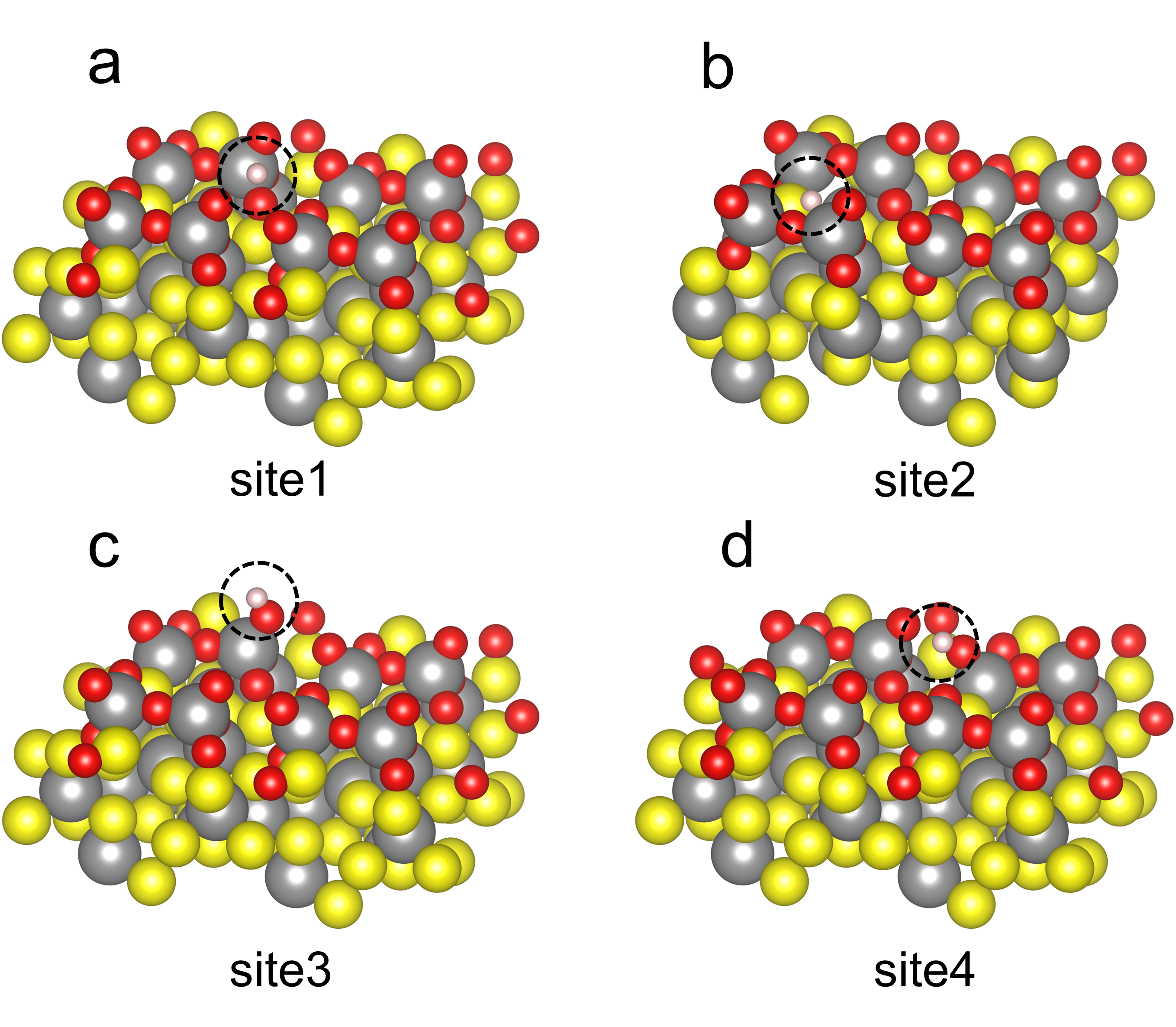


**Figure S23.** Theoretical model of H* adsorption at different sites of V_3_S_4_.


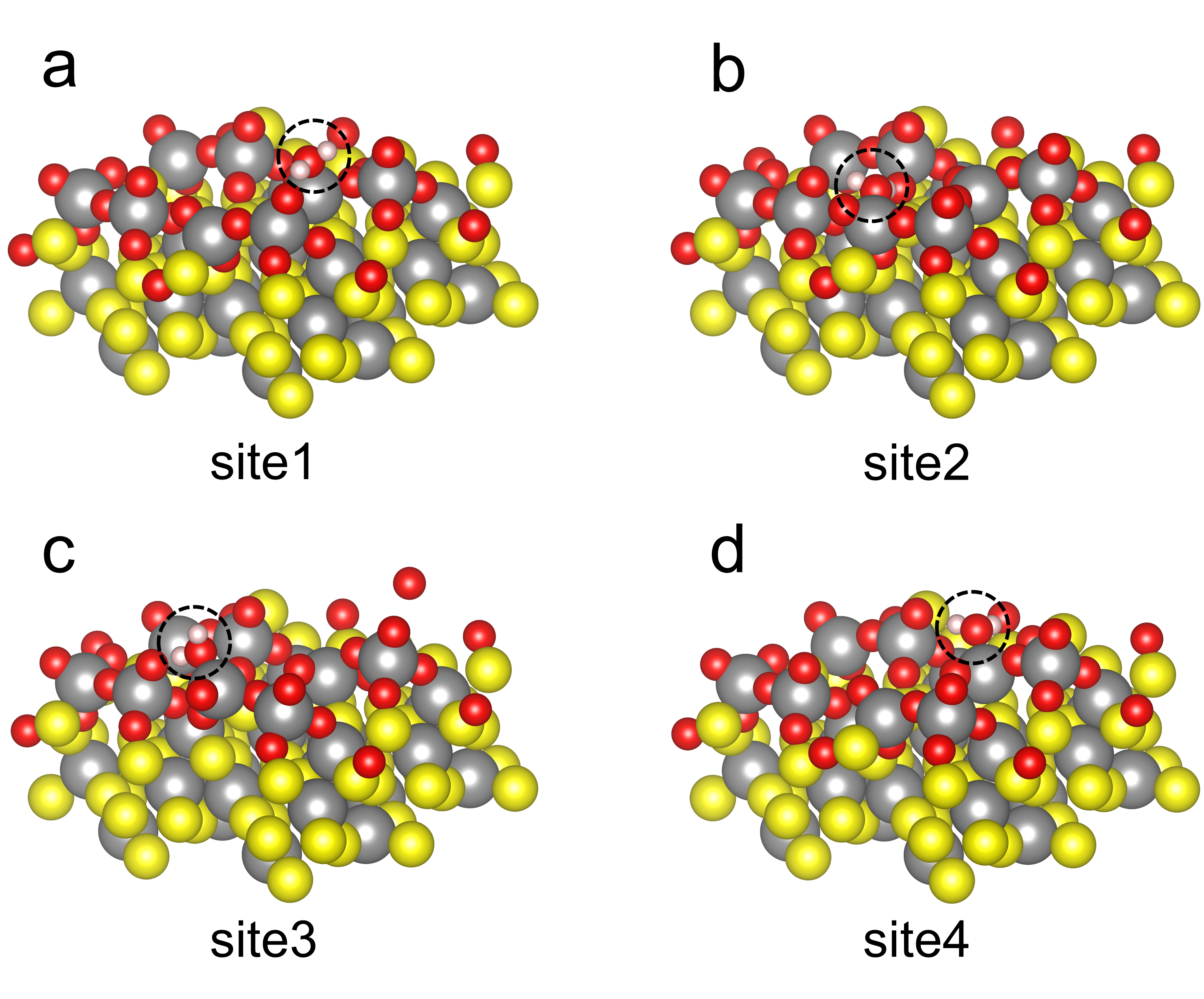


**Figure S24.** Theoretical model of H_2_O adsorption at different sites of V_3_S_4_-Sv.


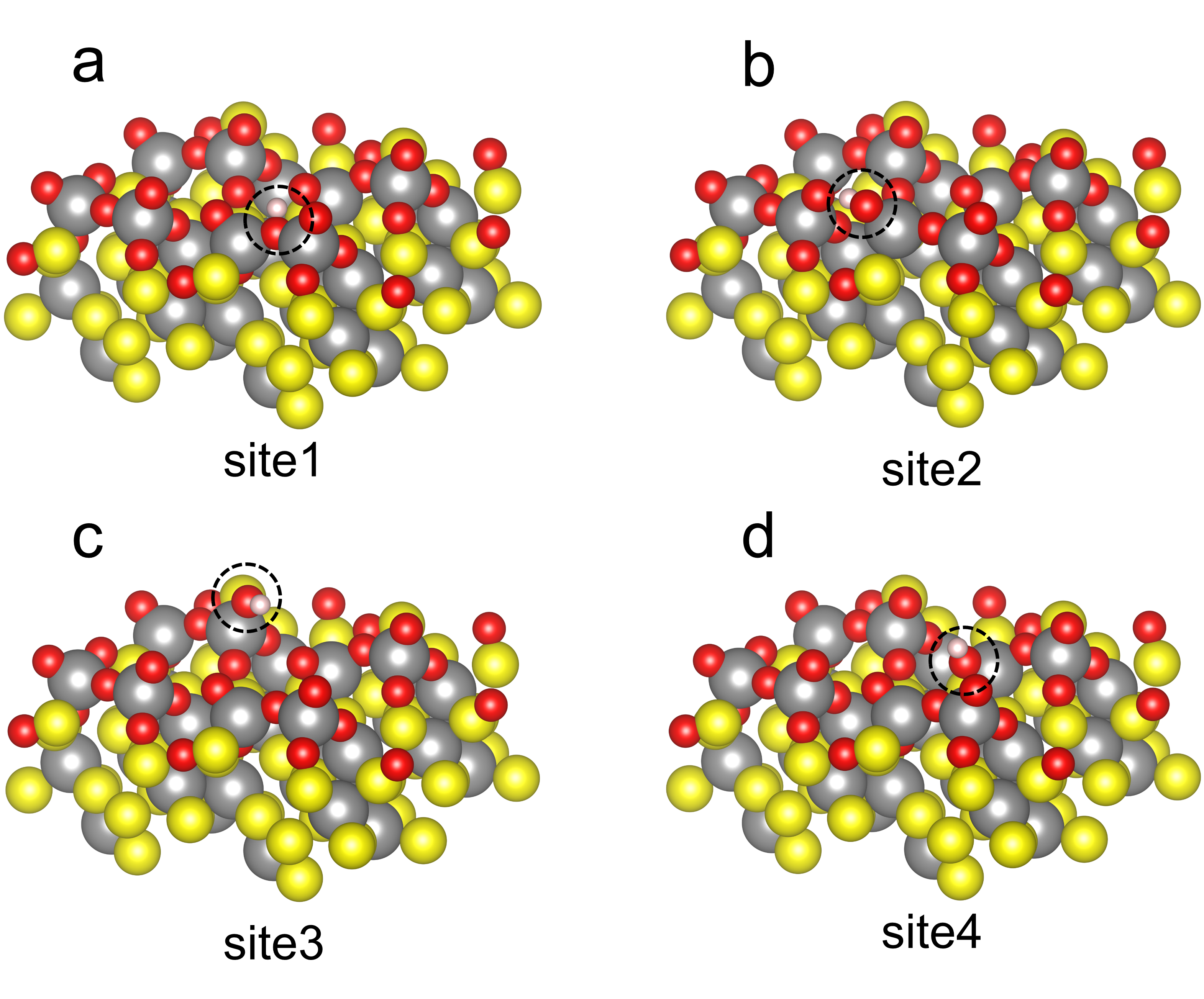


**Figure S25.** Theoretical model of H* adsorption at different sites of V_3_S_4_-Sv.


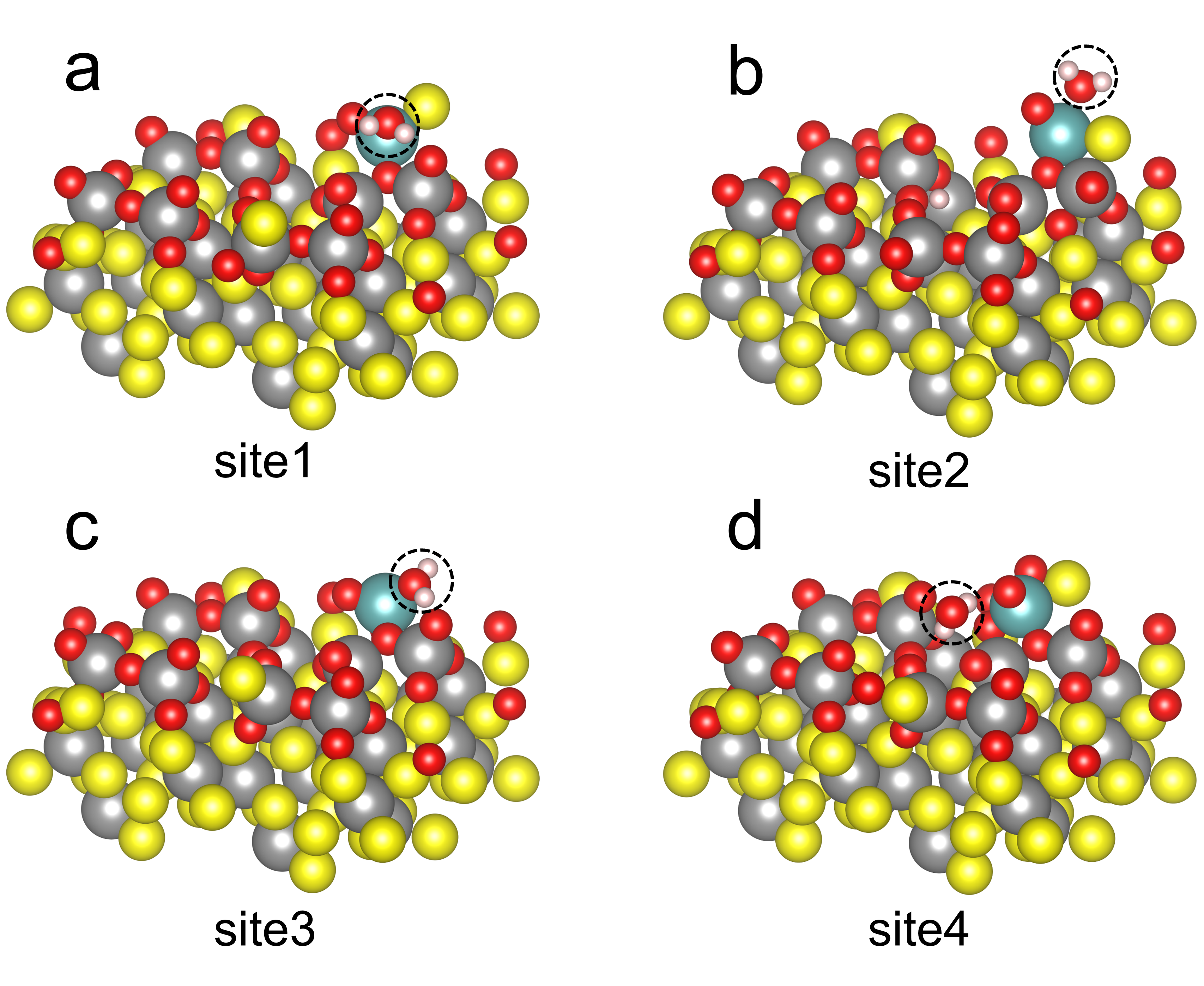


**Figure S26.** Theoretical model of H_2_O adsorption at different sites of W-V_3_S_4_.


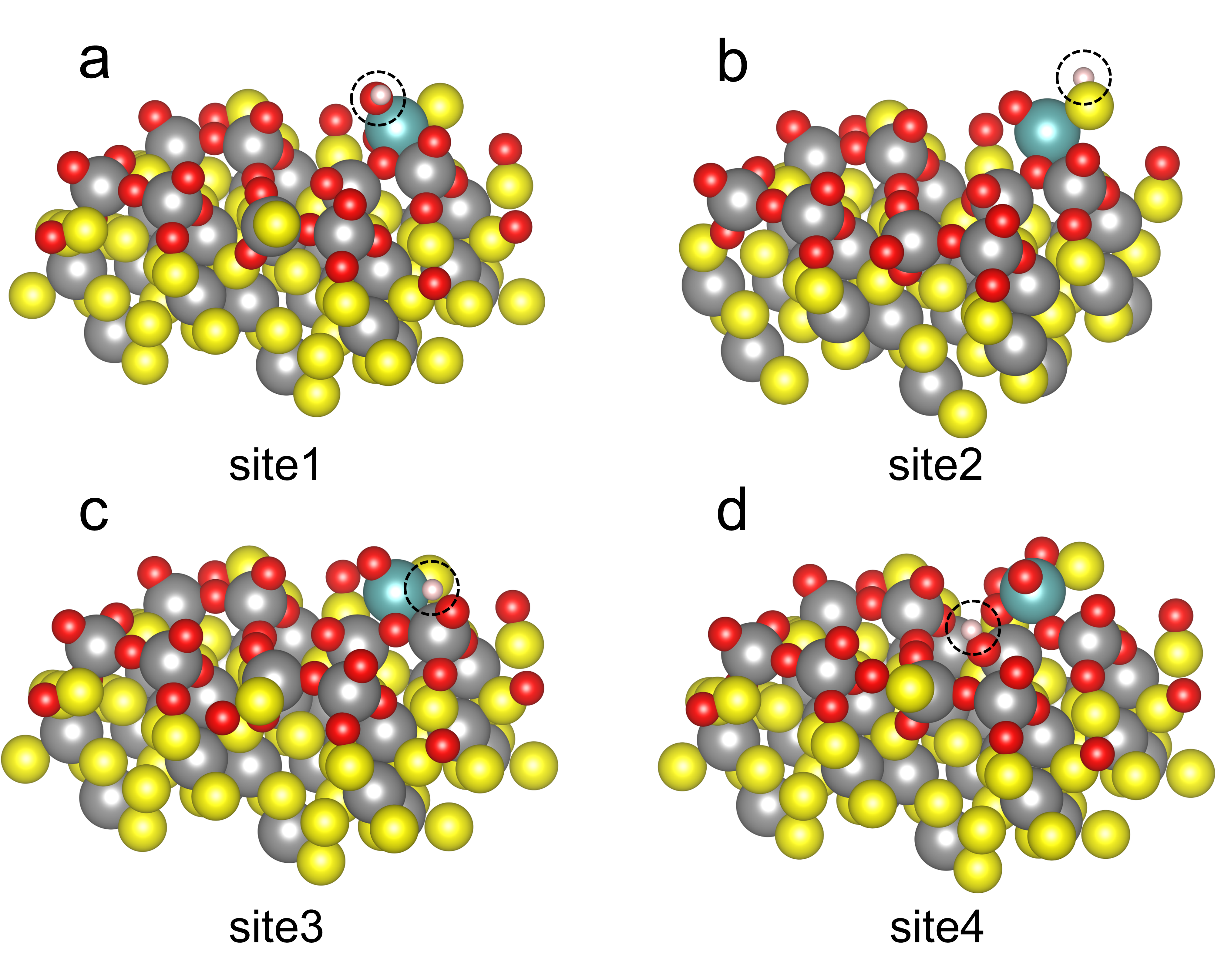


**Figure S27.** Theoretical model of H* adsorption at different sites of W-V_3_S_4_.

**
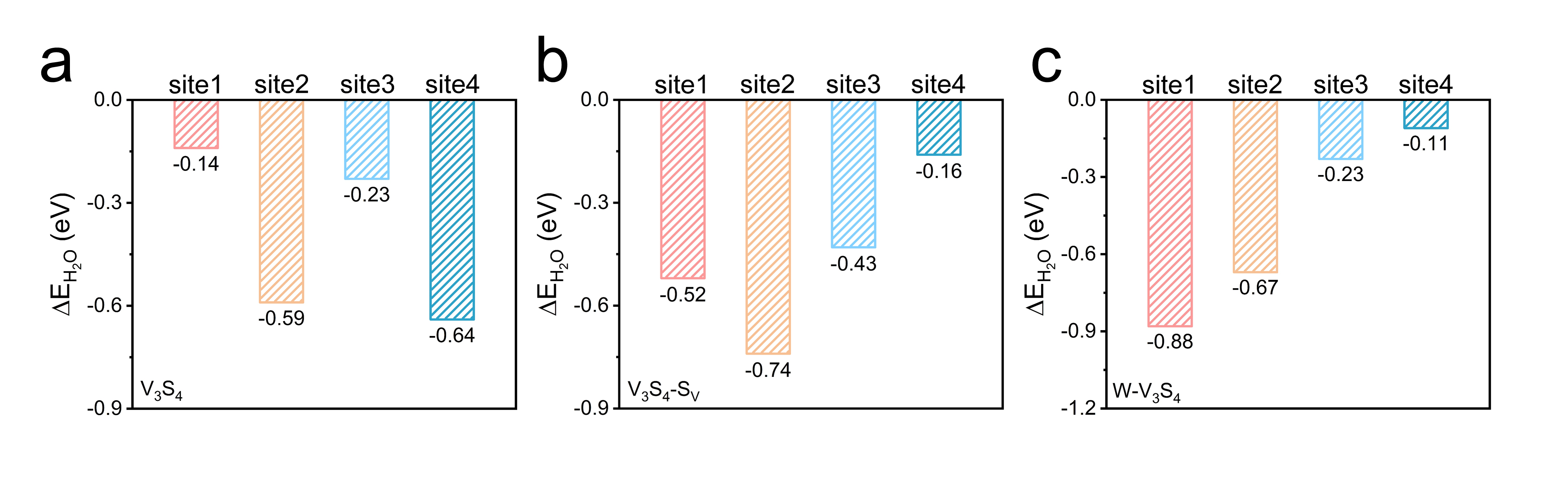
**

**Figure S28.** Calculated H_2_O adsorption energy at different sites of (a)V_3_S_4_, (b)V_3_S_4_-Sv, and (c)W-V_3_S_4_.

**3. Supporting tables**

**Table S1**. Elemental composition of the catalyst material obtained from ICP-OES analysis.

| **Sample**  **W-V_3_S_4_** | **W**  **wt.%** | **V**  **wt.%** | **S**  **wt.%** |
| --- | --- | --- | --- |
| **ICP-OES** | 25.1 | 42.2 | 32.7 |

**Table S2.** Elemental composition of the W-V_3_S_4_ after reaction obtained from ICP-OES analysis.

| **Sample**  **W-V_3_S_4_** | **W**  **wt.%** | **V**  **wt.%** | **S**  **wt.%** |
| --- | --- | --- | --- |
| **ICP-OES** | 21.6 | 52.3 | 26.1 |

**Table S3.** EXAFS fitting parameters at the W L_3_-edge for various samples.

| **Catalysts** | **Path** | **C.N.** | **R (Å)** | **σ^2^ 🞨10^3^ (Å^2^)** | | **ΔE_0_ (eV)** | **R factor** |
| --- | --- | --- | --- | --- | --- | --- | --- |
| W foil | W-W | 8 | 2.72(0.01) | 2.95(0.80) | 7.14(1.53) | | 0.009 |
|  |  | 6 | 3.13(0.01) | 2.95(1.12) |  |  |  |
| W-V_3_S_4_ | W-O | 3.16(0.14) | 1.99(0.02) | 1.98(4.70) | 10.17(3.21) | | 0.012 |
|  | W-S | 1.29(0.97) | 2.33(0.04) | 4.11(1.52) |  |  |  |
|  | W-V | 2.20(0.87) | 3.07(0.02) | 3.51(2.76) |  |  |  |

^a^*N*: coordination numbers; ^b^*R*: bond distance; ^c^*σ^2^*: Debye-Waller factors; ^d^Δ*E*_0_: the inner potential correction. *R* factor: goodness of fit. S_0_^2^, 0.80, was obtained from the experimental EXAFS fitting over W foil reference with known crystallographic value, which was then used to all the samples.

**Table S4.** HER performances of W-V_3_S_4_ and other reported electrocatalysts in the literature.

| **Catalyst** | **Electrolyte** | **η_10_ (mV)**  **(*j*=10mA cm**^−^**^2^)** | **Tafel slope**  **(mV dec**^−^**^1^)** | **Reference** | |
| --- | --- | --- | --- | --- | --- |
| **W-V_3_S_4_** | **1 M KOH** | **54** | **44** | | **This work** |
| Ni_x_Co_1−x_/Ni_3_S_2_@NF | 1 M KOH | 87 | 80 | | 1 |
| Mo-NiS_x_@NiFe LDH/NF | 1 M KOH | 61.3 | 42.3 | | 2 |
| MoCo-VS_2_ | 1 M KOH | 63 | 50 | | 3 |
| Al-CoS_2_ NSs/NF | 1 M KOH | 134 | 79.6 | | 4 |
| NiFe(OH)_x_-Ni_3_S_2_/NF | 1 M KOH | 55 | 46.4 | | 5 |
| Au/Ni_3_S_2_/NF | 1 M KOH | 97 | 72 | | 6 |
| Co-VO_x_-P | 1 M KOH | 98 | 59 | | 7 |
| 1T-Fe/P-WS_2_@CC | 1 M KOH | 116 | 65 | | 8 |
| NiSe_2_-NPs/NiMoN-NRs | 1 M KOH | 58 | 68.7 | | 9 |
| N-WS_2_/Co_3_N | 1 M KOH | 160 | 86 | | 10 |
| Fe_2_P-Co_2_P/NF | 1 M KOH | 65 | 44.4 | | 11 |
| Fe-Ni_3_S_2_/AF | 1 M KOH | 75 | 103 | | 12 |
| W-FeNi_2_S_4_/Ni_3_S_2_/NF | 1 M KOH | 93 | 109.7 | | 13 |
| NiYCe-MOF/NF | 1 M KOH | 136 | 63 | | 14 |
| H-CoS_x_@NiFe LDH/ NF | 1 M KOH | 80 | 90 | | 15 |
| FeCu–BTC/WO_3_–WC | 1 M KOH | 99 | 73.2 | | 16 |
| Mo-NiFeP/NIF | 1 M KOH | 58 | 114 | | 17 |
| N-CoP/CeO_2_ | 1 M KOH | 74 | 47 | | 18 |
| Co-VS_2_/NF | 1 M KOH | 164 | 52 | | 19 |
| N-Ni_3_S_2_/VS_2_ | 1 M KOH | 151 | 107.5 | | 20 |
| P-MoS_2_/CC-300 | 1 M KOH | 81 | 98 | | 21 |
| Ni_3_S_2_@BL MoS_2_ | 1 M KOH | 78.1 | 53.4 | | 22 |
| VS_2_@MoS_2_ | 1 M KOH | 97 | 54.9 | | 23 |
| Nb_4_N_5−x_O_x_-MoS_2_/NG | 1 M KOH | 67 | 44 | | 24 |
| Mo-N/C@MoS | 1 M KOH | 117 | 64.3 | | 25 |
| V-Ni_3_S_2_ | 1 M KOH | 91 | 72.8 | | 26 |

**Table S5.** The Experimentally measured values and the theoretically calculated value of gas.

| Time (min) | Experimentally measured values (mmol) | Theoretically calculated value (mmol) | Faraday efficiency (%) |
| --- | --- | --- | --- |
| 5 | 0.0309 | 0.0311 | 99.36 |
| 10 | 0.0621 | 0.0622 | 99.84 |
| 15 | 0.0931 | 0.0933 | 99.79 |
| 20 | 0.1239 | 0.1244 | 99.60 |
| 25 | 0.1549 | 0.1555 | 99.61 |
| 30 | 0.1824 | 0.1866 | 97.75 |
| 40 | 0.2456 | 0.2487 | 98.75 |
| 50 | 0.3107 | 0.3109 | 99.93 |
| 60 | 0.3730 | 0.3731 | 99.97 |
| Average value (%) | | | 99.40 |

The faradaic efficiency of W-V_3_S_4_ sample was calculated by comparing the amount of experimentally quantified gas (under the current density of 20 mA·cm⁻^2^ by gas chromatography (GC-2014C, Shimadzu) equipped with a thermal conductivity detector for gas (H_2_) quantification) with theoretically calculated gas. η_faradaic_ efficiency = (V_experimental_/V_theoretical_) × 100%. At a constant current density of 20 mA cm⁻^2^, the measured H_2_ values closely matched the calculated values, demonstrating almost 100% faraday efficiency of the assembled electrolyzer.

**Table S6.** Hydrogen adsorption free energy (ΔG_H*_).

| Sites in V_3_S_4_ | *H | Sites in V_3_S_4_-Sv | *H | Sites in W-V_3_S_4_ | *H |
| --- | --- | --- | --- | --- | --- |
| site1 | −0.395 eV | site1 | −1.352 eV | site1 | −0.264 eV |
| site2 | −1.529 eV | site2 | −1.686 eV | site2 | −0.374 eV |
| site3 | −1.820 eV | site3 | −0.819 eV | **site3** | **−0.152** eV |
| site4 | −1.719 eV | site4 | −0.297 eV | site4 | −0.435 eV |

**4. Supporting references**

[1] Z. Wu, Y. Feng, Z. Qin, X. Han, X. Zheng, Y. Deng, W. Hu, *Small* **2022**, *18*, 2106904.

[2] Y. Li, H. Guo, Y. Zhang, H. Zhang, J. Zhao, R. Song, *J. Mater.Chem. A* **2022**, *10*, 18989.

[3] V.K. Singh, U.T. Nakate, P. Bhuyan, J. Chen, D.T. Tran, S. Park, *J. Mater. Chem. A* **2022**, *10*, 9067.

[4] D. Wang, Y. Liu, L. Liu, D. Shan, G. Shen, S. Peng, H. Zhang, X. Wang, *Nano Res.* **2023**, *16*, 6584.

[5] H. Zhang, Y. Zhou, M. Xu, A. Chen, Z. Ni, O. Akdim, T. Wågberg, X. Huang, G. Hu, *ACS Nano* **2022**, *17*, 636.

[6] H. Liu, J. Cheng, W. He, Y. Li, J. Mao, X. Zheng, C. Chen, C. Cui, Q. Hao, *Appl. Catal. B Environ.* **2022**, *304*, 120935.

[7] Z. Zhu, K. Xu, W. Guo, H. Zhang, X. Xiao, M. He, T. Yu, H. Zhao, D. Zhang, T. Yang, *Appl. Catal. B Environ.* **2022**, *304*, 120985.

[8] D.R. Paudel, U.N. Pan, T.I. Singh, C.C. Gudal, N.H. Kim, J.H. Lee, *Appl. Catal. B Environ.* **2021**, *286*, 119897.

[9] J. Wang, D.T. Tran, K. Chang, S. Prabhakaran, D.H. Kim, N.H. Kim, J.H. Lee, *Energy Environ. Mater.* **2022**, *6*, e12526.

[10] L. Liao, Y. Zhao, H. Zhou, D. Li, Y. Qi, Y. Zhang, Y. Sun, Q. Zhou, F. Yu, *Small* **2022**, *18*, 2203171.

[11] H Zhang, H Li, Y Zhou, F Tan, R Dai, X Liu, G Hu, L Jiang, Renbing Wu, *J. Energy Chem.* **2023**, *77*, 420.

[12] M. Wang, L. Zhang, J. Pan, M. Huang, H. Zhu, *Nano Res.* **2021**, *14*, 4740.

[13] J. Jiang, H. Su, S. Song, W. Liu, N. Li, Y. Gao, L. Ge, *Nano Res.* **2023** 11-5974.

[14] F. Li, M. Jiang, C. Lai, H. Xu, K. Zhang, Z. Jin, *Nano Lett.* **2022**, *22*, 7238.

[15] Y.J.L.a.S.-K. Park*, *Small* **2022**, *18*, 2200586.

[16] D.R. Paudel, U.N. Pan, R.B. Ghising, M.R. Kandel, S. Prabhakaran, D.H. Kim, N.H. Kim, J.H. Lee, *Appl. Catal. B Environ.* **2023**, *331*, 122711.

[17] Y. Wang, P. Yang, Y. Gong, D. Liu, S. Liu, W. Xiao, Z. Xiao, Z. Li, Z. Wu, L. Wang, *Chem. Eng. J.* **2023**, *468*, 143833.

[18] L. Zhang, Y. Lei, W. Xu, D. Wang, Y. Zhao, W. Chen, X. Xiang, X. Pang, B. Zhang, H. Shang, *Chem. Eng. J.* **2023**, *460*, 41119.

[19] T. Feng, C. Ouyang, Z. Zhan, T. Lei, P. Yin, Int. J. *Hydrogen Energy* **2022**, *47*, 10646.

[20] X. Zhong, J. Tang, J. Wang, M. Shao, J. Chai, S. Wang, M. Yang, Y. Yang, N. Wang, S. Wang, B. Xu, H. Pan, *Electrochim. Acta*, **2018**, *269*, 55.

[21] J. Tian, C. Yang, R. Hao, F. Li, Z. Liu, W. Chen, Y. Lv, C. Lin, Int. J. *Hydrogen Energy* **2022**, *47*, 17871.

[22] T. Zhang, Y. Liu, J. Yu, Q. Ye, L. Yang, Y. Li, H.J. Fan, *Adv. Mater.* **2022**, *34*, 2202195.

[23] X. Chen, K. Yu, Y. Shen, Y. Feng, Z. Zhu, *ACS Appl. Mater.* **2017**, *9*, 42139.

[24] Y. Yang, Y. Wang, H.-L. He, W. Yan, L. Fang, Y.-B. Zhang, Y. Qin, R. Long, X.-M. Zhang, X. Fan, *ACS Nano* **2020**, *14*, 4925.

[25] I.S. Amiinu, Z. Pu, X. Liu, K.A. Owusu, H.G.R. Monestel, F.O. Boakye, H. Zhang, S. Mu, *Adv. Funct. Mater.* **2017**, *27*, 1702300.

[26] J. Zhou, L. Yu, Q. Zhu, C. Huang, Y. Yu, *J. Mater. Chem. A* **2019**, *7*, 18118.

[27] G. Kresse, J. Furthmuller, *Phys. Rev. B.* **1996**, *54*, 11169.

[28] J. P. Perdew, K. Burke, M. *Phys. Rev. Lett.* **1996**, *77*, 3865-3868.

[29] B. Hammer, L. Hansen, J. K. Norskov, Improved adsorption energetics within density-functional theory using revised Perdew-Burke-Ernzerhof functionals. *Phys. Rev. B* **1999**, *59*, 7413.

[30] H. J. Monkhorst, J. D. Pack, Special points for Brillouin-zone integrations. *Phys. Rev. B* **1976**, *13*, 5188.

[31] Zhang, Y.; Yang, W. Comment on “Generalized gradient approximation made simple. *Phys. Rev. Lett.* **1998**, *80*, 890-890.

[32] G. Henkelman, H. Jónsson, A dimer method for finding saddle points on high dimensional potential surfaces using only first derivatives, *J. Chem. Phys.* **1999**, *111*, 7010.
